# Persistence and Dissipation Pattern of Azoxystrobin and Difenoconazole in Turmeric (*Curcuma longa* L.)

**DOI:** 10.1021/acsomega.6c01375

**Published:** 2026-07-30

**Authors:** Susheel Singh, Lokesh Kumar Saini, Dileswar Nayak, Jyoti Nishad, Chintan Kapadia, Rahul Datta

**Affiliations:** † Food Quality Testing Laboratory, N.M. College of Agriculture, Navsari Agricultural University, Navsari 396450, Gujarat, India; § Department of Plant Molecular Biology and Biotechnology, N.M. College of Agriculture, Navsari Agricultural University, Navsari 396450, Gujarat, India; ∥ Department of Geology and Pedology, Faculty of Forestry and Wood Technology, Mendel University in Brno, Zemedelska 1, 61300 Brno, Czech Republic; ⊥ School of Environmental Sciences, Jawaharlal Nehru University, New Delhi 110067, India

## Abstract

Turmeric (*Curcuma longa* L.) is a major spice crop in India, where foliar diseases such as leaf blotch (*Taphrina maculans*) and leaf spot (*Colletotrichum capsici*) cause substantial yield losses. A ready-mix formulation of azoxystrobin (18.2%) and difenoconazole (11.4%) is effective against these diseases, but no residue data are available for the agro-climatic conditions of South Gujarat. This two-year field study investigated the persistence, dissipation kinetics, and harvest-time residues of both fungicides in turmeric leaves, soil, and rhizomes using a validated QuEChERS-based LC–MS/MS method. First-order kinetics adequately described dissipation, with alternative models (FODE and Higuchi) showing no statistically significant improvement. Azoxystrobin half-lives (DT_50_) ranged from 13.1–14.3 days in leaves and 21.5–21.6 days in soil, while difenoconazole exhibited DT_50_ values of 11.9–12.2 days in leaves and 24.5–25.9 days in soil. The slower soil dissipation of difenoconazole (2.1× longer than in leaves) compared to azoxystrobin (1.6×) is attributed to its higher lipophilicity (log *K*_ow_ 4.4 vs 2.5) and stronger sorption to soil organic carbon (*K*_oc_ 1660–7740 vs 399–1094 mL g^–1^). Residues in turmeric rhizomes at harvest were below the limits of quantification (0.010 mg kg^–1^ for azoxystrobin; 0.005 mg kg^–1^ for difenoconazole) at both recommended and double doses, yielding safety margins exceeding 800-fold relative to established maximum residue limits (8.0 and 4.0 mg kg^–1^, respectively). The extended interval between the last spray and harvest (120–180 days) ensured complete dissipation before rhizome maturity. These findings provide the first baseline residue data for this fungicide combination in turmeric and confirm its safe use under the warm, humid, high-rainfall conditions of South Gujarat.

## Graphical abstract

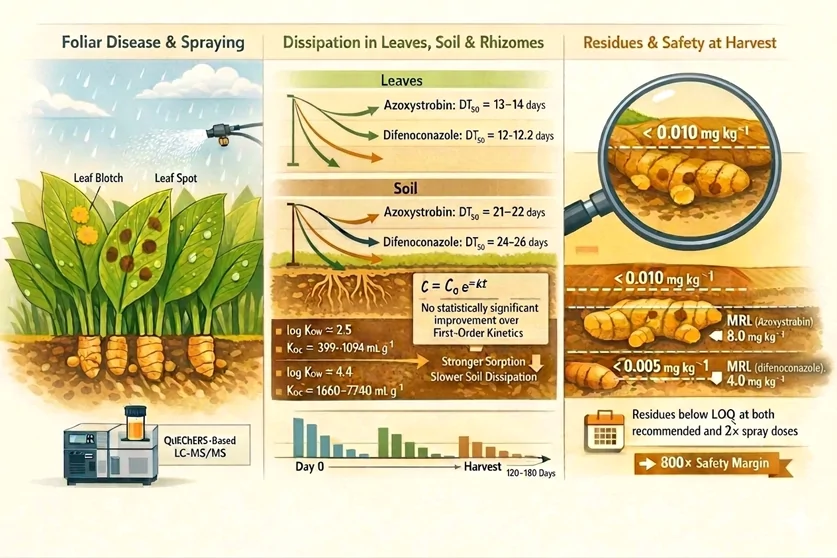

## Introduction

The *Curcuma* genus has a long history of medicinal applications^1,2^ being composed of approximately 133 species.^3^ Among the *Curcuma* species, *Curcuma longa* L. (Curcuma; Turmeric) is the most widely recognized rhizomatous herbaceous perennial medicinal plant belonging to the Zingiberaceae family. It is widely cultivated in India, Sri Lanka, Indonesia, China, Peru, Jamaica, and other tropical and subtropical countries. India is the world’s largest producer of turmeric, accounting for approximately 71% of global production (1.16 million tonnes), followed by Myanmar, China, Nigeria, Bangladesh, and Indonesia as substantially smaller producers.^4^ According to the database of Horticulture Statistics at a Glance, 2017, the turmeric production of India was 1052 thousand MT from an acreage of 193 thousand ha area. Andhra Pradesh, Tamil Nadu, Karnataka, and Gujarat contribute significantly (48.03%) to India’s turmeric production. Gujarat itself cultivated 3711 ha (73148.53 MT yield and 19.71 MT/ha productivity) in 2016–17, with Navsari district accounting for 854 ha (19300.40 MT yield and 22.60 MT/ha productivity). The rhizome, which are short and thick constitute the turmeric of commercial value, composed of a wide variety of compounds, including the bioactive nonvolatile curcuminoids (curcumin, dimethoxy-curcumin, and bisdemethoxy-curcumin) and the compounds present in volatile oil (mono and sesquiterpenoids).^5,6^ It is used as a spice and flavoring agent, for coloring and cosmetic purposes, and is extensively employed in the Indian system of medicine as an ingredient in medicinal oils, pastes, ointments, and other preparations.

The crop is prone to many fungal diseases, leaf blotch (*Taphrina maculans,* Butler) and leaf spot (*Colletotrichum capsici*) [(Syd.), Butler and Bisby] are the most serious foliar diseases resulting in yield and quality losses due to loss of photosynthetic area.^7^ Leaf blotch disease of turmeric, caused by *T. maculans*, was first reported from Rangapur, East Pakistan,^8^ and later found across all turmeric-growing regions. The disease produces numerous leaf spots, more abundant on the upper surface. Spots begin as pale yellow, turning dirty yellow, old gold, or bay shade. They are small (1–2 mm), coalesce readily, and in severe cases, hundreds develop on both sides of leaves. Discrete brownish-black spots are common, especially on lower leaves.^9^ Leaf spot disease of turmeric is caused by *C. capsici*. *Vermicularia curcumae* (Syd.) was the name given by McRae in 1917 to the *C. capsica-*caused turmeric leaf spot disease that was initially discovered in the Coimbatore region of Madras.^10^ It begins with chlorosis on the upper leaf surface, followed by small brown spots that enlarge to 20–30 mm × 5–10 mm. Spots develop grayish centers with reddish margins, and severe infection causes leaf drying.

Leaf blotch and leaf spot are major constraints to turmeric productivity, causing yield losses of 37.6–52.9% and up to 50%, respectively.^11^ Severe infections lead to complete foliage drying, making cultivation uneconomical in some areas, particularly with susceptible varieties.^12^ Warm, rainy, and humid weather favors rapid spread and spore production of the disease, and with susceptible turmeric varieties grown in Gujarat, it poses a serious threat to cultivation. Considering the economic importance of this crop, efforts have been made to evaluate various fungicides for disease management. The ready-mix fungicide azoxystrobin (18.2%) + difenoconazole (11.4%) suspension concentrate (SC) has been found highly effective in reducing foliar diseases of turmeric.^13^ The structures of azoxystrobin and difenoconazole are depicted in Figure 1a,b.

**Figure 1:**
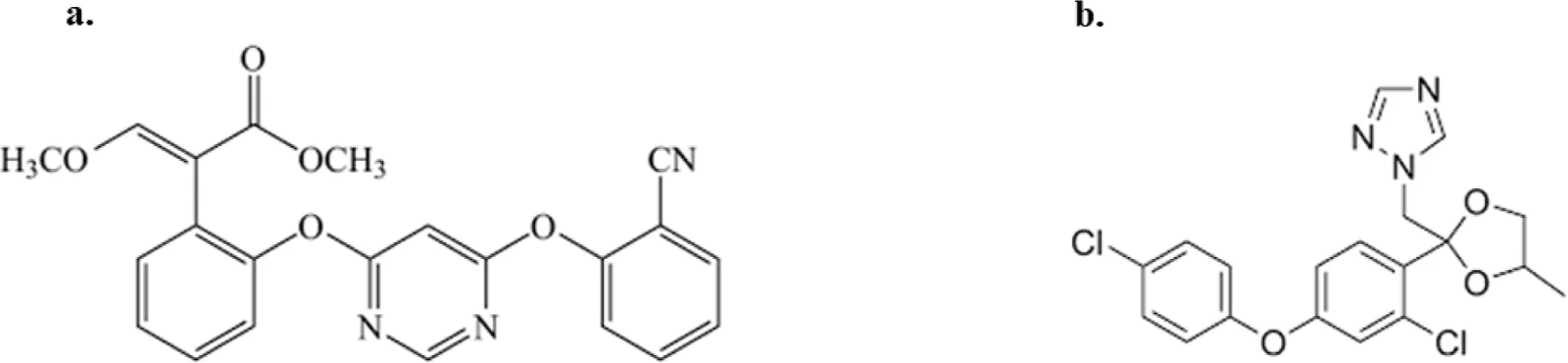
Chemical structure of fungicides (a) azoxystrobin and (b) difenoconazole.

Azoxystrobin (methoxy-acrylate strobilurin; FRAC Group 11) acts by inhibiting electron transfer at complex III of the mitochondrial respiratory chain, thereby blocking ATP synthesis in the fungal cell. Its key physicochemical properties include a log *K*_ow_ of 2.5, water solubility of 6 mg L^–1^, *K*_oc_ = 399–1094 mL g^–1^, and an aerobic soil DT_50_ of 56–179 days under laboratory conditions (FOCUS, 2006).^14^ Difenoconazole (Triazole; FRAC Group 3) inhibits sterol C-14 α-demethylation (DMI), disrupting ergosterol biosynthesis in the fungal cell membrane. Its physicochemical profile differs markedly: log *K*_ow_ = 4.36, water solubility = 15 mg L^–1^, *K*_oc_ = 1660–7740 mL g^–1^, and aerobic soil DT_50_ = 62–126 days (lab).^14^ The higher *K*_oc_ of difenoconazole indicates stronger soil adsorption and reduced bioavailability compared to azoxystrobin, which is expected to translate into greater soil persistence under field conditions. Understanding these intrinsic physicochemical differences is essential for interpreting the dissipation kinetics and environmental fate of the two compounds in the turmeric agro-ecosystem.

Pesticides are integral to agricultural production; however, their overuse contributes to the accumulation of chemical residues, which in turn cause significant environmental degradation and pose substantial risks to human health. At present, information on residue levels of this fungicide mixture is unavailable, both for the state of Gujarat in general and for South Gujarat in particular. This absence constitutes a major knowledge gap with respect to food safety and potential consumer exposure. To bridge this gap, the current investigation was undertaken to study the persistence and dissipation dynamics of azoxystrobin and difenoconazole in turmeric. The results are expected to generate vital evidence to guide safe application practices and ensure effective, yet sustainable, disease management.

## Materials and Methods

### Chemicals and Reagents

Certified reference materials (CRMs) of azoxystrobin and difenoconazole (purity >98%) were purchased from Dr. Ehrenstorfer GmbH (Augsburg, Germany). MS grade acetonitrile and methanol were procured from Merck (Germany). Primary secondary amine (PSA) and anhydrous magnesium sulfate were procured from SUPELCO (Bellefonte, USA). Sodium acetate, sodium chloride (NaCl), and acetic acid were procured from Fisher Scientific (UK). Magnesium sulfate was preheated at 400 °C for 6 h in a muffle furnace and stored in desiccators until use. Milli-Q water was prepared using an ELIX purification system (Merck Millipore).

### Preparation of Standard Solutions

Certified reference materials (CRMs) of azoxystrobin and difenoconazole were weighed precisely using an Aczel analytical balance (Max 60/210 g; *d* = 0.01/0.1 mg) and transferred into 25 mL volumetric flasks. Each compound was first dissolved in acetonitrile, and the final volume was adjusted with the same solvent. Concentrations of the primary standards (*C*_PS_, μg mL^–1^) were calculated according to eq 1:

1 $$C_{\mathrm{PS}}=\frac{m_{\mathrm{CRM}}\times \%P\times 1000}{V_{\mathrm{final}}\times 100}$$

where *m*_CRM_ is the mass of CRM (mg), %*P* is the percent purity, and *V*_final_ is the final volume (mL). From the primary standards, appropriate aliquots were diluted with acetonitrile in 10 mL volumetric flasks to prepare intermediate solutions (20.0 μg mL^–1^). These intermediate solutions were further diluted with acetonitrile in 5 mL volumetric flasks to obtain working standards. Stock, intermediate, and working solutions were stored at −20 ± 4 °C. The stability of the stock solutions was ensured by recording their weight before and after storage, and solvent loss due to evaporation was adjusted gravimetrically.

### Field Experiment

Turmeric (*C. longa* L.) variety “Kesar” was cultivated in field of Department of Natural Resource Management, College of Forestry, Navsari Agricultural University, Gujarat, during the kharif season for two consecutive years of 2022–23 to 2023–24 in 15 m × 10 m plot with row to row spacing at 45 cm and plant to plant spacing at 15 cm. SC of azoxystrobin 18.2% + difenoconazole 11.4% was applied on turmeric leaves with the recommended dose (RD) (150 g a.i. ha^–1^, 10 mL L^–1^) and double RD (300 g a.i/ha^–1^, 20 mL L^–1^), and one plot was maintained as an untreated control, where only water was sprayed. The fungicide was applied three times: the first spray at disease initiation, followed by a second spray 15 days after the first application (DAA), and a third spray 15 DAA after the second application.

### Experimental Site Characterization

The experimental area falls under a tropical climatic regime, receiving heavy monsoon rains, with winters being quite cold and summers fairly hot. Weekly meteorological data were recorded over two consecutive growing seasons (2022–23:45 weeks; 2023–24:43 weeks) from late May to March–April at the experimental site. Maximum temperatures ranged from 27.5–36.4 °C (peaking pre-monsoon, February–June), with minimums ≈11 °C in mid-January. Relative humidity neared saturation (RHmax >98%) in July–August, dropping below 25% by midwinter. Evaporation and sunshine inversely tracked rainfall, minimizing (≤1.0 mm day^–1^; ≤0.3 h day^–1^) during peak monsoon and recovering to 4–6 mm day^–1^ and 8–10 h day^–1^ post-monsoon. Wind speeds averaged 3.7–4.9 km h^–1^, except for a 13.5 km h^–1^ pre-monsoon outlier (June 2023). Rainfall was highly seasonal, concentrated in June–September (total: 2420.3 mm in 2022–23; 1807.7 mm in 2023–24), with weekly maxima of 517 mm both years. Both seasons had 20–26 zero-rain weeks from mid-October (Tables S1.1 and S1.2). The physicochemical properties of the soil samples collected from the experimental site at Navsari feature deep black vertisol soils with a consistent clayey texture (Table S2). Soil pH was slightly alkaline (8.01 and 7.98 in years 1 and 2), EC < 0.5 dS m^–1^ (non-saline), low OC (0.36–0.42%), low available N (204.4–214.1 kg ha^–1^), high P_2_O_5_ (36.2–34.7 kg ha^–1^), high K_2_O (300.3–312.4 kg ha^–1^), and sufficient DTPA-Fe (9.63–10.36 mg kg^–1^). This reflects typical semiarid South Gujarat soils with high buffering and moderate nutrient-holding capacity.

### Sample Collection and Preparation

Approximately 1 kg of soil and 100 g of turmeric leaves were collected from each treatment at 0 (2 h), 1, 3, 5, 7, 10, 15, 20, 30, 40, 50, 60, and 90 days after the final fungicide application, while 500 g of rhizomes was collected at harvest. The samples were sealed in polythene bags and transported to the Food Quality Testing Laboratory of Navsari Agricultural University, Gujarat, India. Sample preparation was carried out on the day of collection, without storage. The leaves and rhizomes were cut into small pieces and homogenized using a heavy-duty variable-speed blender.

### Soil

Residues of difenoconazole and azoxystrobin in soil were determined using the QuEChERS analytical method.^15^ A representative 10 g portion of finely ground soil was transferred in a 50 mL polypropylene centrifuge tube (three replicates), followed by the addition of 4 mL of water and 20 mL of acetonitrile (acidified with 1% acetic acid). The tube was shaken vigorously for 1 min, after which 4 g of magnesium sulfate (MgSO_4_) and 1 g of sodium chloride (NaCl) were added. The mixture was vortexed and centrifuged at 3500 rpm for 2 min. A 10 mL aliquot of the supernatant was transferred to a 15 mL polypropylene centrifuge tube containing 1.5 g of MgSO_4_ and 0.25 g of PSA, then centrifuged again at 3500 rpm for 2 min. The acetonitrile extract (4 mL) was pipetted out in a 25 mL capacity test tube and dried in a low-volume concentrator under nitrogen, and the volume was reconstituted up to 2 mL with methanol: water (80:20) and filtered through a 0.2 μm polytetrafluoroethylene (PTFE) filter into a 2 mL capacity glass vial for quantification on LC–MS/MS.

### Turmeric Leaves and Rhizomes

Residue analysis from turmeric leaves and rhizomes was also carried out using a QuEChERS analytical method.^16^ Each sample was homogenized with a high-speed homogenizer (14,000–15,000 rpm) for 2–3 min. A representative portion of 15.0 ± 0.1 g was transferred to a 50 mL polypropylene centrifuge tube (three replicates), and 15 mL of 1% acetic acid in acetonitrile (v/v) was added. The mixture was shaken vigorously for 2 min and kept at −20 °C for 20–30 min. The tubes containing the samples were treated with 6 g of anhydrous magnesium sulfate and 1.5 g of sodium acetate, mixed thoroughly on a vortex mixer, and centrifuged for 3 min at 3500 rpm. The upper acetonitrile layer (6 mL) was transferred into a 15 mL capacity polypropylene centrifuge tubes containing 0.9 g of MgSO4 and 0.3 g of PSA, followed by vortexing, and was again centrifuged for 2 min at 2500 rpm to get a clean supernatant. The acetonitrile extract (2 mL) was pipetted out into a 25 mL capacity test tube, dried in a low-volume concentrator under nitrogen, reconstituted the volume up to 2 mL with methanol: water (80:20), and filtered through a 0.2 μm PTFE filter into a 2 mL capacity glass vial for quantification on LC–MS/MS.

### LC–MS/MS Analysis

LC–MS/MS analysis was performed using a Thermo Scientific Dionex UltiMate-3000 series UHPLC system coupled to a Thermo Scientific Quantum Access Max triple quadrupole mass spectrometer. Mass spectrometric detection was performed using a Thermo Scientific Quantum Access Max triple quadrupole mass spectrometer equipped with a heated electrospray ionization (HESI) source operating in positive ion mode. The source parameters were optimized as follows: capillary voltage at 4500 V, vaporizer temperature at 350 °C, and capillary temperature at 300 °C, with sheath gas (nitrogen) and auxiliary gas (nitrogen) set at 50 and 10 arbitrary units, respectively. For azoxystrobin, the precursor ion [M + H]^+^ was monitored at *m*/*z* 404.124, with three characteristic product ions at *m*/*z* 329.054 (collision energy 30.73 V), 344.111 (25.12 V), and 372.111 (14.6 V). For difenoconazole, the precursor ion [M + H]^+^ was monitored at *m*/*z* 406.072, with product ions at *m*/*z* 188 (43.72 V), 251 (25.78 V), and 337 (17.38 V). Chromatographic separation was achieved on a Hypersil Gold C18 column (100 × 2.1 mm i.d., 1.9 μm particle size) maintained at ambient temperature. The mobile phase consisted of solvent A (water containing 0.1% formic acid and 5 mM ammonium formate) and solvent B (methanol containing 0.1% formic acid and 5 mM ammonium formate), delivered at a flow rate of 0.3 mL min^–1^ under a gradient elution program: 0–0.5 min at 2% B, 0.5–2.0 min ramped to 40% B, 2.0–10.0 min ramped to 95% B, held at 95% B from 10.0 to 12.0 min, returned to 2% B at 12.1 min, and re-equilibrated until 15.0 min. Under these conditions, azoxystrobin and difenoconazole eluted at retention times (RT) of 10.55 and 12.66 min, respectively, with symmetrical peak shapes and no interfering matrix peaks. The optimized parameters ensured high sensitivity, selectivity, and reproducibility for the quantification of both fungicides in turmeric leaves, soil, and rhizome matrices. Representative chromatograms of both fungicides are shown in Figure 2, along with their corresponding RT.

**Figure 2:**
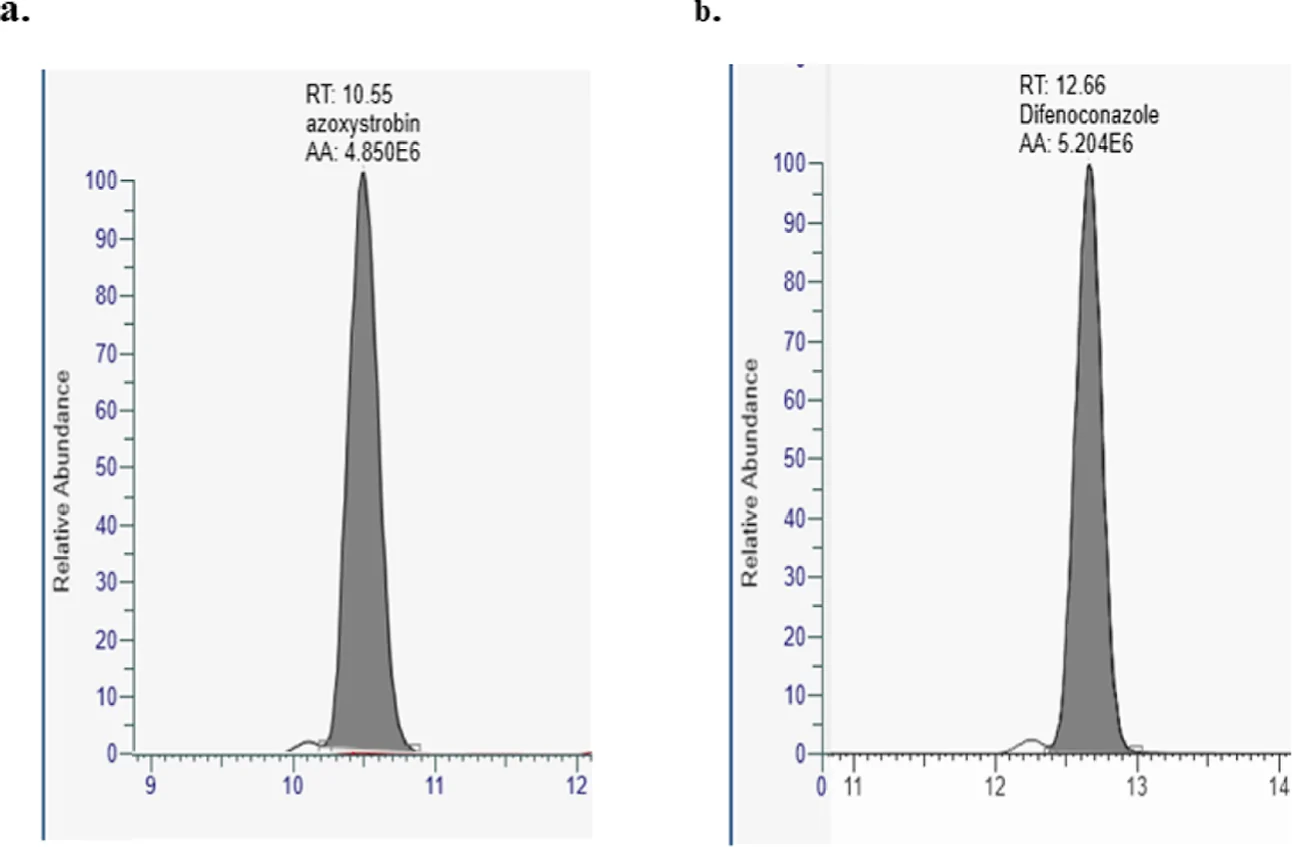
Chromatogram of fungicides on LC–MS/MS (a) azoxystrobin and (b) difenoconazole.

### Method Performance

The suitability of the analytical method was evaluated in accordance with SANTE guidelines,^17^ considering parameters such as accuracy, precision, linearity, selectivity, limit of detection (LOD), and limit of quantification (LOQ). Linearity was assessed using matrix-matched standards to evaluate the performance of the UHPLC-MS/MS system. For this, detector response (peak height/area) was plotted against analyte concentration, and the regression equation along with the coefficient of determination was calculated for the fungicides (Figure 3a,b). Method selectivity was confirmed by the ability to separate and identify each target compound in the presence of other matrix constituents. LOD and LOQ values were estimated based on signal-to-noise ratios of 3 and 10, respectively, using eqs 2 and 3:

2 $$LOD=\frac{3\mathrm{\sigma ̅}}{s_{\mathrm{reg}}}$$

3 LOQ=3.33×LOD

where σ̅ is the mean standard deviation in the response of the standard and *s*_reg_ is the slope of the regression equation.

**Figure 3:**
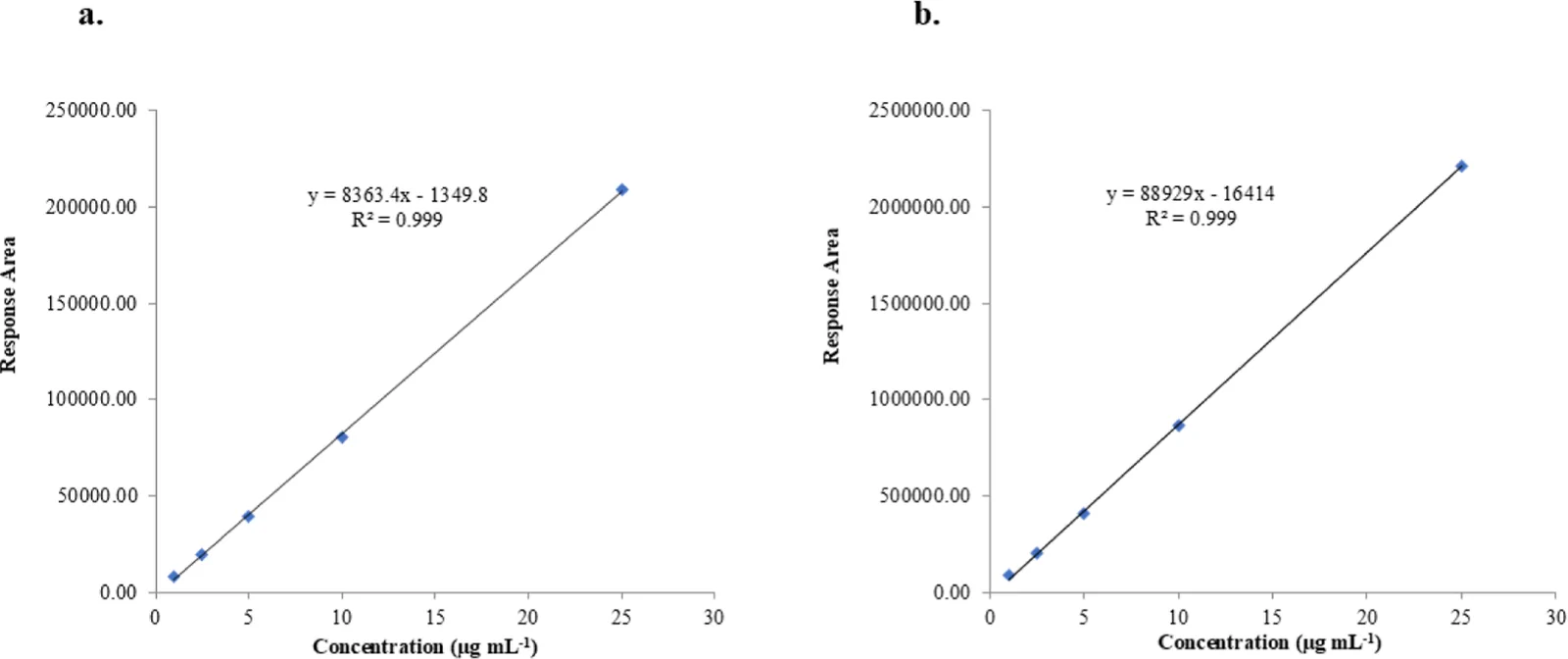
Linearity graph fungicides (a) azoxystrobin and (b) difenoconazole.

Prior to analyzing the test samples from each treatment, a recovery study was performed for leaves, rhizome, and soil to verify the reliability of the analytical method in terms of accuracy (trueness) and precision. According to SANTE guidelines,^17^ percent recovery and relative standard deviation (RSD) serve as indicators of these parameters for quantification of fungicide residues. To evaluate these, representative samples (10 g for soil; 15.0 ± 0.1 g for turmeric leaves and rhizomes) were fortified with difenoconazole and azoxystrobin. The fortification levels were 0.0025, 0.005, and 0.010 mg kg^–1^ for leaf samples, and 0.005, 0.01, and 0.05 mg kg^–1^ for soil and rhizome samples. The fortified samples were maintained at room temperature for 2 h, after which the residues were extracted following the established procedure. To minimize matrix effects, matrix-matched calibration standards were employed for the quantification of fungicide residues. The matrix effect (ME) was calculated by using the following formula:

$$\mathrm{ME}=\left[\frac{{slope}_{matrix}}{{slope}_{solvent}}-1\right]\times 100\%$$

It is being acknowledged that isotopically labeled standards are the best for accurate quantification in mass spectrometry to tackle the matrix effects and sample preparation issues. However, isotopically labeled standards for azoxystrobin and difenoconazole were not used due to high costs. Furthermore, in the present study, the matrix-matched approach was adopted to check matrix effects. Further, it is also acknowledged that the standard addition method was not used in our study, although this quantification approach is commonly used in pesticide residue analysis.

### Data Analysis

#### Kinetic Modeling of Dissipation Data

Prior to performing the kinetic modeling of the residue data, the individual replicate residue values (R1, R2, and R3) from both seasons were combined per time-point, yielding six observations per time-point for each fungicide-matrix combination. Below-quantification-limit (BQL) values were excluded from calculations. The mean (μ) and standard deviation (SD) were computed using

4 $$\mu =\frac{1}{n}\sum_{i}C_{i},SD=\sqrt{\frac{\sum_{i}{\left(C_{i}-\mu \right)}^{2}}{\left(n-1\right)}}$$

The dissipation data of azoxystrobin and difenoconazole in turmeric leaves and soil were fitted to three kinetic models: first-order (FO), first-order double exponential decay (FODED), and Higuchi (square root time) models.

The first-order (FO) model assumes that the rate of dissipation is proportional to the remaining pesticide concentration, yielding a linear relationship between the natural logarithm of residue concentration and time.^14^ This model is expressed as

5 $$C_{t}=C_{0}{\cdot e}^{-kt}$$

where *C_t_* is the residue concentration (mg kg^–1^) at time *t* (days), *C*_0_ is the initial residue concentration, and *k* is the first-order degradation rate constant (day^–1^). The FO model is widely adopted in regulatory risk assessments due to its simplicity.^18^

The FODED model accounts for biphasic dissipation patterns, representing rapid initial dissipation followed by a slower terminal phase. This model is useful when pesticide degradation deviates from simple first-order behavior, such as when a fraction of the residue becomes sequestered or less bioavailable over time.^14,19^ This model is expressed as

6 $$C_{t}=A\cdot e^{-k_{1}t}+{B\cdot e}^{-k_{2}t}$$

where *A* and *B* represent the fractions dissipating at rates *k*_1_ and *k*_2_ (day^–1^), respectively, with *k*_1_ > *k*_2_.

The Higuchi (square root time) model describes dissipation as a function of the square root of time, typically associated with diffusion-controlled release or desorption processes.^20^ This model is expressed as

7 $$C_{t}=C_{0}-a\cdot \sqrt{t}$$

where *a* is the dissipation rate constant (mg kg^–1^ day^–1/2^). The Higuchi model has been applied to describe pesticide release from controlled-release formulations and matrix-limited dissipation.

Model performance was evaluated using the coefficient of determination (*R* ^2^), root-mean-square error (RMSE), Akaike information criterion (AIC), and Bayesian information criterion (BIC). The extra-sum-of-squares F-test was employed to compare the FO and FODED models, with statistical significance set at *p* < 0.05. All kinetic parameters were estimated by nonlinear regression using Microsoft Excel Solver (Microsoft Corporation, Redmond, WA, USA).

The DT_50_ values were calculated from the regression slope (*k*) using the equation DT_50_ = ln(2)/*k*.^21^ The DT_50_ value is defined as the time required for 50% dissipation of the initial pesticide residue, calculated from the first-order rate constant using the relationship DT_50_ = ln(2)/*k*, where *k* is the dissipation rate constant (day^–1^). To quantify the uncertainty associated with the estimated half-lives, 95% confidence intervals for the DT_50_ values were derived using Fieller’s theorem, which accounts for the ratio distribution of ln(2)/*k* and provides statistically robust interval estimates even when the variance of *k* is not negligible.

## Results and Discussion

### Method Performance Validation

The QuEChERS (Quick, Easy, Cheap, Effective, Rugged, and Safe) extraction technique proved suitable for evaluating the method performance parameters in the analysis of fungicides in turmeric leaves, rhizome, and soil (Table 1). Calibration curves for azoxystrobin and difenoconazole were linear across the concentration range of 0.001–0.25 μg mL^–1^, with coefficients of determination (*R* ^2^) ≥ 0.99. The percentage deviation for all tested insecticides remained below 20%. Recovery experiments were performed at fortification levels of 0.0025, 0.005, and 0.010 mg kg^–1^ for leaves sample, and 0.005, 0.01, and 0.05 mg kg^–1^ for soil and rhizome samples, showing recoveries within the acceptable range (70–120%) and relative standard deviations (RSDs) below 20% for leaves, soil, and rhizome matrices (Table 1 ). For azoxystrobin, recoveries ranged from 74.85–86.37% in leaves, 78.39–86.34% in soil, and 78.34–89.95% in rhizomes, whereas for difenoconazole, they were 87.58–100.35%, 89.20–105.80%, and 88.56–95.59%, respectively. The RSD values for azoxystrobin varied between 7.79–9.10% in leaves, 9.25–15.23% in soil, and 7.45–10.39% in rhizomes, while for difenoconazole, they ranged from 4.12–7.27%, 8.47–16.60%, and 10.33–12.18%, respectively. The limits of detection (LOD) and limit of quantification (LOQ) in matrix-matched standards were 0.005 and 0.010 mg kg^–1^ for azoxystrobin, and 0.002 and 0.005 mg kg^–1^ for difenoconazole, respectively. Indian FSSAI/CIB&RC maximum residue limits (MRLs) for this compound–crop combination have not yet been established. Therefore, United States Environmental Protection Agency (USEPA) establishes tolerances, which are functionally equivalent to MRLs, were considered in our study. The US EPA tolerance for azoxystrobin in turmeric (Subgroup 1C) is 8.0 mg/kg and for difenoconazole is 4.0 mg/kg.^22,23^ The LOQ for both fungicides in turmeric rhizomes (0.010 mg kg^–1^ for azoxystrobin; 0.005 mg kg^–1^ for difenoconazole) was well below the corresponding US EPA tolerances (8.0 and 4.0 mg kg^–1^, respectively). Matrix effects (ME) were evaluated by comparing the slopes of matrix-matched calibration curves prepared in turmeric leaves, soil, and turmeric rhizome extracts against solvent-based calibration curves. Calibration curves were constructed over a concentration range of 0.001–0.025 μg mL^–1^ for both analytes. For azoxystrobin, the matrix effect in turmeric leaves was +13.07%, indicating mild ion enhancement. In soil, a slight suppression of −7.31% was observed, which is within the acceptable ±20% limit per SANTE guidelines.^17^ However, turmeric rhizome exhibited moderate ion suppression of −17.18%, approaching the ±20% threshold. For difenoconazole, similar trends were observed. Turmeric leaves showed mild enhancement (+10.44%), while soil (−20.45%) and turmeric rhizome (−19.47%) exhibited moderate ion suppression, with soil just exceeding the ±20% limit. Although the matrix effect for difenoconazole in soil (−20.45%) marginally exceeded the SANTE-recommended threshold of ±20%, the use of matrix-matched calibration standards effectively compensated for this suppression, as evidenced by acceptable recovery values (89.20–105.80%) and RSDs (<20%) for soil matrix.

**Table 1:** Method Performance Verification Studies of Azoxystrobin and Difenoconazole in Soil and Turmeric Leaves and Rhizomes^a^

S. no.	Parameter	Particular			Azoxystrobin	Difenoconazole
1	Linearity (*n* = 5)	Calibration concentration range			0.001–0.025 μg mL^–1^	0.001–0.025 μg mL^–1^
		Regression equation			*y* = 8363.4*x* – 1349.8	*Y* = 88929*x* – 16414
		*R* ^2^ {*R* ^2^ ≥ 0.99}			0.999	0.999
2	Sensitivity (*n* = 5)	LOD (mg kg^–1^)			0.005	0.002
		LOQ (mg kg^–1^) {<MRL}			0.010	0.005
		MRL (mg kg^–1^)			8.0	4.0
3	Matrix Effect	Calibration concentration range			0.001–0.025 μg mL^–1^	0.001–0.025 μg mL^–1^
		Regression equation (Matrix-Leaves)			*y* = 9456.3*x* – 1568	*y* = 98215*x* + 27415
		Regression equation (Matrix-Soil)			*y* = 8765*x* + 3541	*y* = 78129*x* – 14675
		Regression equation (Matrix-Rhizome)			*y* = 7259*x* – 2745	*y* = 64528*x* – 24596
		ME (%)-Leaves			13.07	10.44
		ME (%)-Soil			–7.31	–20.45
		ME (%)-Turmeric Rhizome			–17.18	–19.47
4	Accuracy (*n* = 7)	% Recovery {(70–120%}	Matrix	F level (mg kg^–1^)	(%)	(%)
			Leaves	0.0025	74.85 ± 5.83	87.58 ± 5.59
				0.005	81.23 ± 5.90	100.35 ± 2.14
				0.001	86.37 ± 8.86	96.75 ± 8.83
			Soil	0.005	82.35 ± 7.48	93.68 ± 7.23
				0.01	78.39 ± 5.63	89.20 ± 9.17
				0.05	86.34 ± 4.95	105.8 ± 11.28
			Rhizome	0.005	78.34 ± 9.60	92.93 ± 7.42
				0.01	89.95 ± 4.27	88.56 ± 5.29
				0.05	85.69 ± 7.23	95.59 ± 8.96
5	Precision (*n* = 7)	% RSD {≤20%}	Matrix	F level (mg kg^–1^)	(%)	(%)
			Leaves	0.0025	9.10	4.12
				0.005	7.79	6.39
				0.001	8.87	7.27
			Soil	0.005	12.42	10.33
				0.01	15.23	8.47
				0.05	9.25	16.60
			Rhizome	0.005	10.39	10.33
				0.01	7.45	11.25
				0.05	10.29	12.18

a n: number of replications; *R* ^2^: coefficient of determination; LOQ: limit of quantification; MRL: maximum residue limit; F level: fortification level; RSD: relative standard deviation; and values given in parentheses { } are the standard acceptance criteria as per SANTE guidelines.^17^

### Validation of Kinetic Models

Prior to the dissipation study and determining the kinetics of Fungicides, the residue data obtained in the study was pooled, and each data set was fitted to first-order (FO), first-order double exponential (FODE), and Higuchi (square root time). The comparison of kinetic models for the dissipation of azoxystrobin and difenoconazole in turmeric leaves and soil is presented in Table 2. To validate the assumption of first-order (FO) kinetics, three competing models, viz., FO, FODE, and Higuchi (square root time), were fitted to all eight data sets (two fungicides × two doses × two matrices). The FO model consistently outperformed the Higuchi model across all data sets, yielding higher coefficients of determination (*R* ^2^), lower root-mean-square errors (RMSE), and lower Akaike (AIC) and Bayesian (BIC) information criteria. The poorest fit was observed for the Higuchi model applied to difenoconazole in soil at the RD (*R* ^2^ = 0.765), confirming that the square root time model is inappropriate for these data. While the FODE model showed marginally higher *R* ^2^ values (ranging from 0.856 to 0.948) compared to FO (0.825 to 0.946), this improvement came at the cost of two additional parameters. The extra-sum-of-squares F-test revealed no statistically significant improvement of FODE over FO for any data set (*p* > 0.05 in all cases).

**Table 2:** Comparison of Kinetic Models for Dissipation of Azoxystrobin and Difenoconazole in Turmeric Leaves and Soil (Pooled 2022–2024)^a^

Matrix	Compound	Dose	Model	Equation	*k* ± SE (day^–1^)	*R* ^2^	RMSE	AIC	BIC	χ^2^ (*p* value)
**Leaves**	Azoxystrobin	RD	FO	*C* = 0.131·e^–0.053*t*^	0.053 ± 0.007	0.902	0.021	38.2	39.8	0.23
			FODE	*C* = 0.152·e^–0.048*t*^ + 0.037·e^–0.002*t*^	0.048, 0.002	0.948	0.019	40.1	42.9	0.31
			Higuchi	*C* = 0.189 – 0.031·√t	—	0.887	0.031	44.5	46.1	0.08
**Leaves**	Azoxystrobin	2X RD	FO	*C* = 0.263·e^–0.049*t*^	0.049 ± 0.005	0.927	0.028	41.5	43.1	0.18
			FODE	*C* = 0.268·e^–0.045*t*^ + 0.051·e^–0.001*t*^	0.045, 0.001	0.931	0.026	43.2	46.0	0.26
			Higuchi	*C* = 0.319 – 0.041·√t	—	0.865	0.037	47.1	48.7	0.06
**Leaves**	Difenoconazole	RD	FO	*C* = 0.161·e^–0.057*t*^	0.057 ± 0.008	0.825	0.025	40.8	42.4	0.15
			FODE	*C* = 0.221·e^–0.051*t*^ + 0.047·e^–0.003*t*^	0.051, 0.003	0.928	0.023	42.9	45.7	0.22
			Higuchi	*C* = 0.268 – 0.035·√t	—	0.859	0.033	46.2	47.8	0.07
**Leaves**	Difenoconazole	2X RD	FO	*C* = 0.246·e^–0.058*t*^	0.058 ± 0.007	0.897	0.032	44.1	45.7	0.20
			FODE	*C* = 0.301·e^–0.023*t*^ + 0.074·e^–0.001*t*^	0.023, 0.001	0.914	0.030	46.3	49.1	0.28
			Higuchi	*C* = 0.375 – 0.044·√t	—	0.848	0.040	49.8	51.4	0.05
**Soil**	Azoxystrobin	RD	FO	*C* = 0.148·e^–0.0321*t*^	0.032 ± 0.004	0.876	0.018	39.5	41.1	0.12
			FODE	*C* = 0.198·e^–0.028*t*^} + 0.047·e^–0.001*t*^	0.028, 0.001	0.896	0.017	41.3	44.1	0.17
			Higuchi	*C* = 0.245 – 0.019·√t	—	0.823	0.024	44.2	45.8	0.05
**Soil**	Azoxystrobin	2X RD	FO	*C* = 0.284·e^–0.0322*t*^	0.032 ± 0.003	0.928	0.022	42.0	43.6	0.14
			FODE	*C* = 0.312·e^–0.028*t*^ + 0.077·e^–0.001*t*^	0.028, 0.001	0.901	0.020	44.1	46.9	0.19
			Higuchi	*C* = 0.389 – 0.028·√t	—	0.831	0.029	47.3	48.9	0.06
**Soil**	Difenoconazole	RD	FO	*C* = 0.042·e^–0.027*t*^	0.027 ± 0.002	0.946	0.009	35.2	36.8	0.09
			FODE	*C* = 0.042·e^0.048*t*^ + 0.016·e^–0.002*t*^	0.048, 0.002	0.856	0.008	37.4	40.2	0.13
			Higuchi	*C* = 0.058 – 0.008·√t	—	0.765	0.011	40.1	41.7	0.04
**Soil**	Difenoconazole	2X RD	FO	*C* = 0.072·e^–0.028*t*^	0.028 ± 0.003	0.945	0.014	38.8	40.4	0.11
			FODE	*C* = 0.069·e^–0.024*t*^ + 0.019·e^–0.001*t*^	0.024, 0.001	0.883	0.013	40.9	43.7	0.16
			Higuchi	*C* = 0.088 – 0.011·√t	—	0.802	0.017	43.5	45.1	0.05

a FO = first-order; FODE = first-order double exponential; SE = standard error; RMSE = root-mean-square error; AIC = Akaike information criterion; and BIC = Bayesian information criterion. The Higuchi model does not produce a rate constant (k) in the same sense as FO/FODE; the parameter shown is the slope (a).

The complete goodness-of-fit statistics for the first-order kinetic model, including chi-square (χ^2^), root-mean-square error (RMSE), and AIC values for each fungicide-matrix-dose combination, are provided in Table 2. In brief, χ^2^ values ranged from 0.004 to 0.152 (*p* ≥ 0.999 for all data sets), RMSE values from 0.009 to 0.032 mg kg^–1^, and AIC values from 35.2 to 44.1. The consistently low RMSE values, near-unity p-values, and favorable AIC comparisons confirm that the first-order kinetic model provides a statistically adequate fit for all data sets, with no evidence of lack-of-fit.

Consequently, guided by the principle of parsimony and supported by the model comparison statistics (AIC, BIC, and F-test), the first-order kinetic model was selected as the most parsimonious and statistically robust description of the dissipation kinetics for both fungicides in turmeric leaves and soil under the agro-climatic conditions of South Gujarat.

### Residues, Dissipation, and Persistence Study

The residues of azoxystrobin and difenoconazole in turmeric leaves, soil, and rhizomes under the agro-climatic conditions of South Gujarat are presented in Tables 3 and 4 and year wise data is presented in Tables S3.1–S3.4. The residue data from two consecutive years (2022–23 and 2023–24) were pooled to determine the dissipation patterns of azoxystrobin and difenoconazole adopting FO degradation kinetics in turmeric leaves and soil following application at recommended dose (RD = 150 g a.i. ha^–1^) and double RD (2X RD = 300 g a.i. ha^–1^) (Table 5). Student’s *t*-test performed, which revealed no significant difference in DT_50_ between years for any treatment (*p* > 0.05 in all cases, range 0.181–0.884; Table S4), confirming that pooling of two-year data was statistically justified. The limits of quantification (LOQ) was 0.010 mg kg^–1^ for azoxystrobin and 0.005 mg kg^–1^ for difenoconazole. Residues were quantified at intervals from 0 (2 h) to 90 days after the final application. Figure 4 depicts a 3D plot of the first-order dissipation kinetics of azoxystrobin and difenoconazole in leaves and soil at the recommended and double the RD based on pooled residues data (2 years). It also displays the exponential decay curve onto the *XY*-floor and the linearized form ln­(*C*) = ln­(*C*_0_) – *kt* onto the *XZ* back-plane simultaneously for better visualization. It should be noted that the *Z*-axis is not an independent dimension; rather, it is mathematically defined as *Z* = ln­(Y), a direct transformation of the *Y*-axis (residue).

**Table 3:** Residue (mg/Kg) and Dissipation (%) of Azoxystrobin in Turmeric Leaves, Soil, and Rhizome (Pooled 2 Years)

	Azoxystrobin residues (mg/kg)					
	Turmeric leaves		Soil		Rhizome	
Day	RD	2X RD	RD	2X RD	RD	2X RD
0 (2 h)	0.136 ± 0.002 (—)	0.298 ± 0.007 (—)	0.238 ± 0.011(—)	0.386 ± 0.006(—)	–	–
1	0.129 ± 0.004 (5.1%)^a^	0.278 ± 0.010 (6.7%)	0.226 ± 0.013 (5.0%)	0.367 ± 0.011 (4.9%)	–	–
3	0.112 ± 0.004 (17.6%)	0.256 ± 0.008 (14.1%)	0.189 ± 0.012 (20.6%)	0.339 ± 0.018 (12.2%)	–	
5	0.099 ± 0.003 (27.2%)	0.224 ± 0.014 (24.8%)	0.112 ± 0.015 (52.9%)	0.245 ± 0.007 (36.5%)	–	–
7	0.075 ± 0.008 (44.9%)	0.183 ± 0.006 (38.6%)	0.087 ± 0.008 (63.4%)	0.217 ± 0.013 (43.8%)	–	–
10	0.049 ± 0.010 (64.0%)	0.157 ± 0.006 (47.3%)	0.081 ± 0.008 (66.0%)	0.149 ± 0.016 (61.4%)	–	–
15	0.032 ± 0.010 (76.5%)	0.087 ± 0.005 (70.8%)	0.076 ± 0.008 (68.1%)	0.131 ± 0.013 (66.1%)	–	–
30	0.015 ± 0.004 (89.0%)	0.041 ± 0.007 (86.2%)	0.030 ± 0.011 (87.4%)	0.072 ± 0.008 (81.3%)	–	–
60	**BQL (<0.010)** **(>92.6%)**	0.020 ± 0.008 (93.3%)	0.019 ± 0.003 (92.0%)	0.040 ± 0.004 (89.6%)	–	–
90	— (—)	**BQL (<0.010)** (**>96.6%)**	0.012 ± 0.003 (95.0%)	0.021 ± 0.003 (94.6%)	–	–
At Harvest	BQL	**BQL**	BQL	BQL	BQL	**BQL**
BQL	(0.010 mg kg^–1^)					

a Figures in parentheses represent % dissipation.

**Table 4:** Residue and Dissipation (%) of Difenoconazole in Turmeric Leaves, Soil, and Rhizome (Pooled 2 Years)

	Difenoconazole residues (mg/kg)					
	Turmeric leaves		Soil		Rhizome	
Day	RD	2X RD	RD	2X RD	RD	2X RD
0 (2 h)	0.261 ± 0.016 (—)	0.363 ± 0.029 (—)	0.054 ± 0.013 (—)	0.085 ± 0.012 (—)	–	–
1	0.180 ± 0.014 (31.0%)^a^	0.292 ± 0.023 (19.6%)	0.046 ± 0.006 (14.8%)	0.078 ± 0.008 (8.2%)	–	–
3	0.170 ± 0.011 (34.9%)	0.263 ± 0.024 (27.5%)	0.043 ± 0.006 (20.4%)	0.072 ± 0.010 (15.3%)	–	–
5	0.162 ± 0.011 (37.9%)	0.245 ± 0.019 (32.5%)	0.041 ± 0.006 (24.1%)	0.067 ± 0.011 (21.2%)	–	–
7	0.147 ± 0.010 (43.7%)	0.172 ± 0.047 (52.6%)	0.032 ± 0.005 (40.7%)	0.060 ± 0.010 (29.4%)	–	–
10	0.051 ± 0.012 (80.5%)	0.099 ± 0.028 ()72.7%)	0.028 ± 0.005 (48.1%)	0.053 ± 0.011 (37.6%)	–	–
15	0.029 ± 0.005 (88.9%)	0.069 ± 0.024 (81.0%)	0.023 ± 0.005 (57.4%)	0.038 ± 0.004 (55.3%)	–	–
30	0.018 ± 0.008 (93.1%)	0.033 ± 0.011 (90.9%)	0.019 ± 0.005 (64.8%)	0.027 ± 0.003 (68.2%)	–	–
60	0.008 ± 0.002 (96.9%)	0.013 ± 0.003 (96.4%)	0.009 ± 0.002 (83.3%)	0.015 ± 0.003 (82.4%)	–	–
90	**BQL (<0.005) (>98.1%)**	**BQL (<0.005) (>98.6%)**	**BQL (<0.005) (>90.7%)**	**BQL (<0.005) (>94.1%)**	BQL	BQL
BQL	(0.005 mg kg^–1^)					

a Figures in parentheses represent % dissipation.

**Table 5:** First-Order Dissipation Kinetics of Azoxystrobin and Difenoconazole in Turmeric Leaves and Soil under Field Conditions (Pooled Over 2 Years)^a^

Fungicide	Dose	Matrix	Regression equation	*k* ± SE (day^–1^)	*R* ^2^	DT_50_ (days)^b^	95% CI
Azoxystrobin	RD	Leaves	*C* = 0.131·e^–0.053*t*^	0.053 ± 0.007	0.902	13.1	9.2–16.8
Azoxystrobin	2X RD	Leaves	*C* = 0.263·e^–0.049*t*^	0.049 ± 0.005	0.927	14.3	10.7–17.8
Azoxystrobin	RD	Soil	*C* = 0.148·e^–0.0321*t*^	0.0321 ± 0.004	0.876	21.6^a^	14.9–28.2
Azoxystrobin	2X RD	Soil	*C* = 0.284·e^–0.0322*t*^	0.0322 ± 0.003	0.928	21.5^a^	16.6–26.3
Difenoconazole	RD	Leaves	*C* = 0.161·e^–0.057*t*^	0.057 ± 0.008	0.825	12.2	7.2–17.2
Difenoconazole	2X RD	Leaves	*C* = 0.246·e^–0.058*t*^	0.058 ± 0.007	0.897	11.9	8.3–15.5
Difenoconazole	RD	Soil	*C* = 0.042·e^–0.027*t*^	0.027 ± 0.002	0.946	25.9	20.3–31.4
Difenoconazole	2X RD	Soil	*C* = 0.072·e^–0.028*t*^	0.028 ± 0.003	0.945	24.5	19.2–29.7

a RD = recommended dose (150 g a.i. ha^–1^); 2X RD = double recommended dose (300 g a.i. ha^–1^). *C* = residue concentration (mg kg^–1^) at time *t* (days); *k* = first-order degradation rate constant; SE = standard error. DT_50_ = half-life calculated as ln(2)/*k*; 95% confidence intervals derived using Fieller’s theorem.

b The *k* values for azoxystrobin in soil (0.0321 and 0.0322 day^–1^ at RD and 2X RD, respectively) are rounded to 0.032 in the table; DT_50_ values are calculated from the unrounded regression outputs.

**Figure 4:**
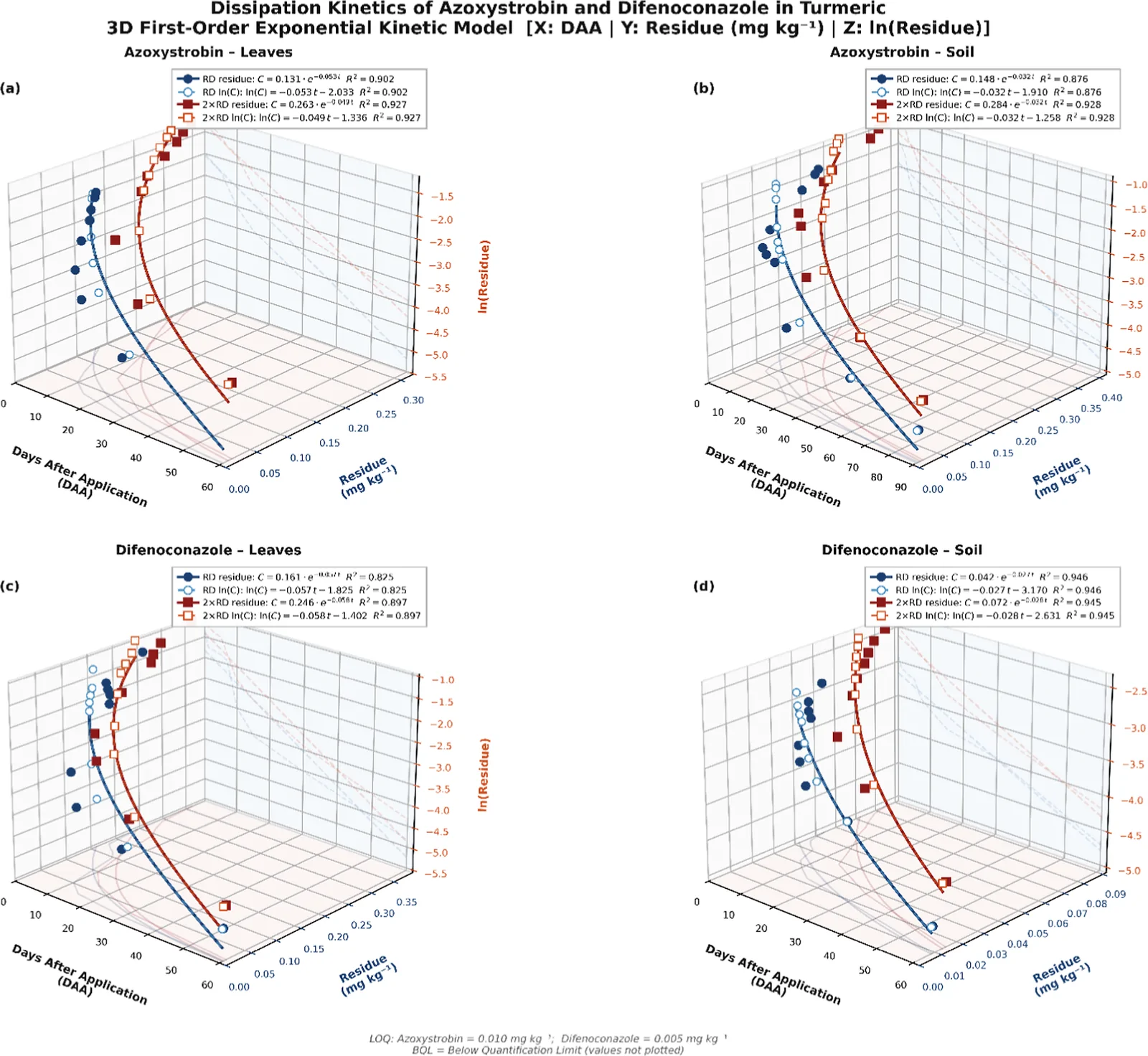
3D plot of the first-order dissipation kinetics of azoxystrobin and difenoconazole in leaves and soil at the recommended and double the RD based on pooled residues data (2 years).

### Azoxystrobin

#### Turmeric Leaves

The initial pooled residues recorded on day 0 (2.0 h) were 0.136 mg kg^–1^ at the RD and 0.298 mg kg^–1^ at the double RD (2X RD). Residues declined rapidly within the first 7 days (44.9% at RD and 38.6% at 2X RD), followed by steady dissipation to 89.0% (RD) and 86.2% (2X RD) by day 30. At RD, residues fell below the LOQ (0.010 mg kg^–1^) by day 60 (>92.6% dissipation), while at 2X RD, residues remained quantifiable (0.020 mg kg^–1^) at day 60 but declined below LOQ by the 90th day. The first-order rate constants (*k*) were 0.053 day^–1^ (RD) and 0.049 day^–1^ (2X RD), corresponding to half-lives (DT_50_) of 13.1 days and 14.3 days, respectively, confirming dose-independent dissipation behavior. After 90th day, the residue declined to below LOQ (0.010 mg kg^–1^) in leaves at both RD and 2X RD, which were less than the United States MRL 8.0 mg kg^–1^.^22^ The *R* ^2^ values for foliar dissipation (0.902 and 0.927) indicate an excellent fit of the first-order model, explaining over 90% of the variance in residue concentrations over time.

The relatively rapid dissipation of azoxystrobin on turmeric leaves (DT_50_ ≈ 13–14 days) arises from agro-climatic conditions during the 2022–23 and 2023–24 cropping seasons in South Gujarat. Foliar exposure to solar radiation (3.8 to 6.2 h day^–1^ monsoon, 8.0 to 10.0 h thereafter) drives photodegradation, a primary pathway for strobilurins. Warm temperatures (mean 26.2 to 26.4 °C, max 27.5 to 36.4 °C) coupled with high humidity (>98% monsoon) enhance volatilization and microbial activity on leaf surfaces. Intense rainfall (2420.3 mm in 2022–23, 1807.7 mm in 2023–24, weekly max 517 mm) causes substantial wash-off of surface residues. Rapid leaf expansion further dilutes concentrations through plant growth. Collectively, elevated rainfall, temperature, humidity, and insolation accelerate azoxystrobin decline on turmeric foliage.

#### Soil

Azoxystrobin dissipation in soil was markedly slower than in leaves, with initial pooled residues of 0.238 mg kg^–1^ (RD) and 0.386 mg kg^–1^ (2X RD). Soil exhibited a slower initial decline (5.0% loss by day 1 at RD), followed by an accelerated phase between days 3 and 7 (52.9% loss by day 5 at RD). This progressive dissipation trend resulted in 87.4% (RD) and 81.3% (2X RD) loss by day 30, while residues remained detectable at day 90 (0.012 mg kg^–1^ at RD; 0.021 mg kg^–1^ at 2X RD), representing 95.0% and 94.6% dissipation, respectively. Neither dose reached the LOQ (0.010 mg kg^–1^) within 90 days. The first-order rate constant was 0.032 day^–1^ for both doses, yielding half-lives of 21.6 days (RD) and 21.5 days (2X RD). The *R* ^2^ values for soil dissipation (0.876 and 0.928) indicate an acceptable to excellent fit. Azoxystrobin dissipated more slowly in soil (DT_50_ 21.6 days) than on leaves (13.1 days). Soil blocks photodegrade because it receives little direct sunlight. Microbes primarily cause soil dissipation, depending on soil traits. The soil was clayey, slightly alkaline (pH 7.98–8.01), non-saline (EC 0.32–0.35 dS m^–1^), with moderate organic carbon (0.36–0.42%) and good nutrients (N: 204–214 kg ha^–1^; P: 35–36 kg ha^–1^; K: 300–312 kg ha^–1^). These traits support active microbes, but azoxystrobin’s moderate lipophilicity (log *K*_ow_ 2.5) lets it bind loosely to soil organic carbon. This binding reduces the amount that microbes can access and slows breakdown, so residues last longer in soil.

Azoxystrobin dissipated approximately 1.6 times faster on leaves (DT_50_ = 13.1–14.3 days) than in soil (DT_50_ = 21.5–21.6 days), with the 95% confidence intervals showing only marginal overlap, confirming statistically significant matrix-dependent persistence. Dissipation behavior was independent of application dose, as rate constants at RD and 2X RD were statistically indistinguishable (leaves: 0.053 vs 0.049 day^–1^; soil: 0.032 day^–1^ for both), confirming that half-life is an intrinsic property independent of initial concentration.

The DT_50_ values obtained (13–22 days) are consistent with literature reports. Faster dissipation in tomato fruits (DT_50_ = 1.82–2.32 days)^24^ reflects higher water content and metabolic activity, while values in mango fruits (10.4–12.1 days)^25^ align closely with our leaf findings. Banasiak et al. (2011)^26^ reported soil half-lives of 3–22 days; our value of 21.5–21.6 days falls at the higher end, consistent with the warm, humid conditions of South Gujarat.

The longer persistence in turmeric compared to tomato may be attributed to azoxystrobin’s moderate lipophilicity (log *K*_ow_ 2.5) and high *K*_oc_ (399–1094 mL g^–1^), which reduce bioavailability through sorption to soil organic matter. Additionally, the dense, waxy cuticle of turmeric leaves may limit photolytic degradation and wash-off, further extending foliar persistence.

### Difenoconazole

#### Turmeric Leaves

Difenoconazole dissipation in turmeric leaves exhibited a multiphasic pattern: rapid initial loss, a plateau, followed by accelerated decline. Initial pooled residues were 0.261 mg kg^–1^ (RD) and 0.363 mg kg^–1^ (2X RD), approximately 1.4 times higher at the elevated dose. By day 1, residues declined by 31.0% (RD) and 19.6% (2X RD), indicating rapid volatilization and photodegradation. Between days 1 and 7, residues plateaued (31.0–43.7% dissipation at RD), suggesting absorption into leaf tissues. Dissipation then accelerated dramatically between days 7 and 10, reaching 80.5% (RD) and 72.7% (2X RD) by day 10. By day 30, residues were 0.018 mg kg^–1^ (RD) and 0.033 mg kg^–1^ (2X RD) (93.1% and 90.9% dissipation), declining further to 0.008 mg kg^–1^ and 0.013 mg kg^–1^ by day 60. Both doses fell below the LOQ (0.005 mg kg^–1^) by day 90, which were less than the United States MRL 4.0 mg kg^–1^.^23^ The first-order rate constants (0.057 day^–1^ at RD; 0.058 day^–1^ at 2X RD) yielded half-lives of 12.2 days and 11.9 days, respectively, confirming dose-independent kinetics despite the observed multiphasic pattern.

The *R* ^2^ values for difenoconazole on leaves were 0.825 at the RD and 0.897 at the double dose, which indicates an acceptable to good fit of the first-order model. The lower value at the RD is still acceptable for field studies because: (1) the LOQ is very low (0.005 mg kg^–1^), so near this limit measurements become less precise; (2) difenoconazole is absorbed into leaves due to its systemic nature, making its movement more complex. The fit improves at the double dose because higher initial residues stay above the LOQ longer, reducing measurement error. Since difenoconazole dissipation in turmeric has not been previously reported, the half-life values determined in this study were evaluated against those documented in other crops to facilitate interpretation of the results. For instance, in apples, difenoconazole half-life ranged from 12 to 21 days, with residues declining to 0.01 mg kg^–1^ within 50–79 days post-application.^27^ Difenoconazole has shown longer persistence in some citrus fruits (DT_50_ ≈ 19.2 days in clementine mandarins), reflecting matrix effects and environmental conditions.^28^ Additionally, studies in tea leaves have shown a half-life of 1.77 days.^29^ These comparisons suggest that difenoconazole persistence is influenced by crop type, environmental conditions, and application rates.

#### Soil

Difenoconazole dissipation in soil followed a steady and consistent decline, unlike the multiphasic pattern observed in leaves. Initial pooled residues were 0.054 mg kg^–1^ (RD) and 0.085 mg kg^–1^ (2X RD), approximately 1.6 times higher at the elevated dose. By day 7, dissipation reached 40.7% (RD) and 29.4% (2X RD), progressing to 57.4% (RD) and 55.3% (2X RD) by day 15, 64.8% (RD) and 68.2% (2X RD) by day 30, and 83.3% (RD) and 82.4% (2X RD) by day 60. Both doses fell below the LOQ (0.005 mg kg^–1^) by day 90. The first-order rate constants were 0.027 day^–1^ (RD) and 0.028 day^–1^ (2X RD), with corresponding half-lives of 25.9 days and 24.5 days, confirming dose-independent kinetics. The soil half-life was approximately 2.1 times longer than in leaves (12.2 days at RD), highlighting pronounced matrix-dependent persistence. Difenoconazole exhibited a pronounced matrix effect, with soil half-lives (24.5–25.9 days) approximately 2.1 times longer than in leaves (11.9–12.2 days), more pronounced than for azoxystrobin (1.6×). This is attributable to difenoconazole’s higher lipophilicity (log *K*_ow_ 4.4 vs 2.5) and higher *K*_oc_ (1660–7740 mL g^–1^), promoting stronger sorption to the clayey, slightly alkaline soil (pH 7.98–8.01; organic carbon 0.36–0.42%). Our soil half-life falls at the lower end of the EFSA-reported range (20–120 days), indicating relatively rapid dissipation under the warm, humid conditions of South Gujarat (mean temperature 26.2–26.4 °C; rainfall 1807.7–2420.3 mm; RH > 98% during monsoon). While heavy rainfall and warm temperatures support microbial activity, strong sorption to soil organic carbon reduces bioavailability, resulting in moderate persistence. These physicochemical and environmental interactions explain the observed dissipation kinetics in the turmeric agro-ecosystem.

### Mechanistic Basis for Faster Foliar Dissipation of Azoxystrobin and Difenoconazole

The faster dissipation of both fungicides on turmeric leaves compared with soil can be mechanistically explained by the simultaneous action of several abiotic and biotic pathways on the foliar surface, which are largely restricted or completely absent in the soil environment.

Photolysis is a primary driver of foliar dissipation. The leaf surface in our experimental site (South Gujarat) receives direct solar radiation, with average sunshine hours of approximately 4.0 h/day during the monsoon (Table S1.2) and increasing to 7.5 h/day post-monsoon (Tables S1.1 and S1.2). As reported by Ghosh and Singh,^30^ azoxystrobin degrades faster under UV light than under sunlight; correspondingly, Man et al.^31^ showed that difenoconazole generates multiple photolysis transformation products, confirming its susceptibility to UV-driven degradation. The leaf surface is also a site of active surface hydrolysis. The warm and moist foliar microenvironment during the monsoon season (RH frequently exceeding 95%, with maximum humidity reaching 98.4% in some weeks; Table S1.1) and mean temperature averaging 27.9 °C (Tables S1.1 and S1.2) facilitates aqueous-phase hydrolysis of both fungicides, which was experimentally demonstrated for difenoconazole in water.^31^ Moreover, intense rainfall (total seasonal rainfall: 2420.3 mm in 2022–23, 1807.7 mm in 2023–24, with weekly maxima of 517 mm; Tables S1.1 and S1.2) physically removes surface residues via wash-off, a mechanism that is essentially absent for soil-adsorbed residues. A modeling study on apple orchards performed by An et al.,^32^ for example, attributed 29.8–75.8% of the decline in pesticide concentration to fruit growth dilution, underscoring the importance of the dilution pathway. Warm temperatures also promote volatilization of surface residues, and rapid leaf expansion during the monsoon season causes growth dilution, both of which contribute to the apparent decline in leaf concentrations.

In contrast, dissipation in soil is governed predominantly by microbial degradation and is tightly controlled by sorption–desorption equilibria. The experimental soil is a deep black vertisol (clayey texture, pH 7.98–8.01, EC < 0.5 dS/m, organic carbon 0.36–0.42%; Table S2). Even with moderate organic carbon, the clay-dominated matrix promotes adsorption of both fungicides [(especially the more lipophilic difenoconazole (log *K*_ow_ 4.36, *K*_oc_ 1660–7740 mL g^–1^)] compared with azoxystrobin (log *K*_ow_ 2.5, *K*_oc_ 399–1094 mL g^–1^; FOCUS^14^). Paszko and Jankowska^33^ demonstrated that stronger sorption of triazole fungicides reduces bioavailability, thereby limiting microbial access and slowing degradation, which might be expected to be more pronounced for difenoconazole. A detailed degradation study of difenoconazole in soil by Man et al.^31^ further identified four transformation products via photolysis, hydrolysis, and soil degradation, confirming that microbial breakdown is the dominant process in the subsurface. Photodegradation is effectively excluded in the soil environment because UV radiation penetrates only the uppermost 1–2 mm of the soil surface; moreover, residues are distributed deeper after irrigation and rainfall, further limiting photolytic breakdown. Consequently, microbial degradation, which depends on soil moisture, temperature, aeration, pH, and organic matter quality, becomes the primary but slower dissipation pathway.

Thus, the 1.6–2.1 times faster dissipation of both fungicides on leaves than in soil (Table 5) cannot be attributed to a single factor but to the combined effect of several. On leaves, photolysis, surface hydrolysis, rainfall wash-off, volatilization, and growth dilution simultaneously accelerate dissipation. In contrast, limited organic carbon reduces microbial degradation in soil, and clay particles bind the fungicides (particularly difenoconazole), which further slows breakdown. Moreover, soil lacks the breakdown processes that happen on leaves, where direct sunlight and surface moisture work together to degrade fungicides. Difenoconazole showed a greater difference because it adheres more strongly to soil organic matter and clay.^14^

### Rhizomes

The harvest-time residue analysis revealed that both azoxystrobin and difenoconazole residues in turmeric rhizomes were below the respective limits of quantification (0.010 mg kg^–1^ and 0.005 mg kg^–1^) at both recommended and double recommended doses (Tables 3 and 4), and its chromatogram is shown in Figure S5. This finding is consistent with the observed dissipation kinetics, which showed complete foliar dissipation by day 60–90 and soil dissipation by day 90–120, depending on the compound and application rate. Importantly, turmeric rhizomes are harvested approximately 210–270 days after planting, with the final fungicide application occurring around 90–100 days after planting. Consequently, the interval between the last spray and harvest ranged from 120 to 180 days, a period substantially longer than the observed DT_50_ values (12–26 days) and the time required for residues to decline below the LOQ (60–90 days). This extended preharvest interval provides a substantial margin of safety, ensuring complete dissipation of residues from both foliar and soil matrices before rhizome maturity.

Furthermore, based on the regression equations in Table 5, the calculation of preharvest intervals (PHI; time for residues to reach the US EPA tolerances of 8.0 mg kg^–1^ for azoxystrobin and 4.0 mg kg^–1^ for difenoconazole from the initial deposit) are not meaningful for leaves and soil, since initial deposits are already far below these tolerances.^22,23^ The operational PHI (time to fall below LOQ) was approximately 60 days (azoxystrobin RD, leaves), 90 days (azoxystrobin double dose, leaves), and 90 days (difenoconazole both doses, leaves and soil), which is substantially shorter than the actual last-spray-to-harvest interval of 120–180 days. The absence of detectable residues in the edible portion, combined with safety margins exceeding 800-fold relative to the established US EPA tolerances (8.0 mg kg^–1^ for azoxystrobin and 4.0 mg kg^–1^ for difenoconazole),^22,23^ confirms that the application of this azoxystrobin–difenoconazole combination at the recommended schedule (three sprays at 15 day intervals) poses no detectable consumer safety risk under the agro-climatic conditions of South Gujarat. These data support the safe and judicious use of this fungicide formulation for the management of foliar diseases in turmeric cultivation. Consistent with these findings, similar observations were reported in earlier studies, where residues of the ready-mix formulation of azoxystrobin and difenoconazole were detected below the LOQ levels in soil collected at harvest from rice and tomato fields.^34,35^

## Conclusion

This study provides the first comprehensive assessment of the persistence, dissipation kinetics, and harvest-time residues of the azoxystrobin–difenoconazole combination in turmeric (*C. longa* L.) under the agro-climatic conditions of South Gujarat. The validated QuEChERS-based LC–MS/MS method demonstrated satisfactory linearity (*R* ^2^ ≥ 0.99), recovery (74.85–105.80%), and precision (RSD < 20%) for both fungicides in leaves, soil, and rhizomes, with LOQ values of 0.010 mg kg^–1^ (azoxystrobin) and 0.005 mg kg^–1^ (difenoconazole).

Kinetic modeling across eight data sets confirmed that first-order (FO) kinetics adequately described dissipation, with alternative models (FODE and Higuchi) showing no statistically significant improvement (F-test *p* > 0.05). Azoxystrobin dissipated 1.6 times faster in leaves (DT_50_ = 13.1–14.3 days) than in soil (DT_50_ = 21.5–21.6 days). Difenoconazole exhibited a more pronounced matrix effect, with soil half-lives (24.5–25.9 days) approximately 2.1 times longer than leaf half-lives (11.9–12.2 days). This difference is attributed to difenoconazole’s higher lipophilicity (log *K*_ow_ 4.4 vs 2.5) and greater sorption to soil organic carbon (*K*_oc_ 1660–7740 vs 399–1094 mL g^–1^). Both fungicides exhibited dose-independent dissipation, confirming that half-life is an intrinsic property independent of initial concentration. Critically, residues in turmeric rhizomes at harvest were below the LOQ for both fungicides at both application rates. The interval between the final spray and harvest (120–180 days) substantially exceeded the observed DT_50_ values and the time required for residues to fall below the LOQ (60–90 days). Residues were below LOQ at harvest, substantially lower than established tolerances, indicating a favorable residue profile. This finding provides a basis for regulatory consideration but does not constitute a formal dietary risk assessment. However, the safety margins exceeded 800-fold relative to established MRLs (8.0 mg kg^–1^) for azoxystrobin; 4.0 mg kg^–1^ for difenoconazole for turmeric confirming negligible consumer risk. Future studies could explore the effects of different environmental conditions, crop varieties, and integrated pest management strategies on fungicide persistence, to further optimize safe and sustainable use in turmeric cultivation along with elucidating the metabolic fate of both fungicides in turmeric plants using radiolabeled or high-resolution mass spectrometry studies; investigating the effects of different soil types, organic amendments, and irrigation regimes on dissipation kinetics; evaluating the potential for accumulation under multiyear continuous cropping; and conducting dietary risk assessments using probabilistic modeling approaches. Nevertheless, the present data establish a robust scientific basis for the safe and effective use of this fungicide combination in turmeric cultivation under the warm, humid, high-rainfall condition characteristic of South Gujarat.

## Limitations and Future Directions

The present study has several limitations that should be considered when interpreting the findings. First, residue analysis in turmeric rhizomes was conducted only at harvest, due to the destructive nature of rhizome sampling and the requirement of the CIB&RC residue trial protocol for rhizome crops to specify harvest-time sampling for the edible portion. While the finding of residues below the LOQ at harvest is reassuring from a consumer risk perspective, intermediate rhizome sampling at 30, 60, and 90 days post-application (ideally using a parallel-plot design) would have provided more complete information on systemic acropetal movement and temporal accumulation dynamics; this is recommended for future studies. Second, the higher χ^2^-error values in several data sets (particularly azoxystrobin in soil at RD and difenoconazole in leaves) indicate that the SFO model provides only a moderate fit in these cases; biphasic kinetic models (e.g., DFOP) could be explored in future studies with higher sampling frequency, particularly in the early monsoon period. Third, the absence of microbial biomass or enzyme activity measurements limits mechanistic interpretation of the soil dissipation data. Fourth, all data were generated from the Navsari agro-climatic zone, and extrapolation to other turmeric-growing regions of India with distinct climatic and soil profiles should be made with caution. In addition to this, another limitation of this study was that metabolites of azoxystrobin and difenoconazole were not quantified. The metabolite analysis aligned with EFSA/Codex residue definitions should be pursued in future studies, particularly if data are to be submitted to support MRL applications in the EU or global markets as well for better understanding the environmental fate of these compounds.

## Supplementary Material



## Notes

Weekly meteorological data for the 2022–23 growing season at the experimental site (Navsari, Gujarat, India); weekly meteorological data for the 2023–24 growing season at the experimental site; physicochemical properties of soil samples collected from the experimental site at Navsari Agricultural University; year-wise residue data (mg kg^–1^) for azoxystrobin and difenoconazole in turmeric leaves, soil, and rhizomes for 2022–23 and 2023–24 separately; student’s *t*-test results comparing DT_50_ values between year 1 (2022–23) and year 2 (2023–24) for all treatment combinations; and representative LC–MS/MS chromatograms of azoxystrobin and difenoconazole in turmeric rhizomes at harvest, confirming residues below the LOQ (PDF)
