# Supplementary material for: Persistence and Dissipation Pattern of Azoxystrobin and Difenoconazole in Turmeric (Curcuma longa L.)
Source: ACS Omega. 2026 Jul 30;11(32):47485–97. doi: 10.1021/acsomega.6c01375 (PMC13491983; doi:10.1021/acsomega.6c01375)
Supplement: Supplementary file 1 [file ao6c01375_si_001.pdf]

Persistence and Dissipation Pattern of Azoxystrobin and Difenoconazole in Turmeric (*Curcuma longa* L.)

Susheel Singh[1], Lokesh Kumar Saini[1], Dileshwar Nayak[2], Jyoti Nishad[1], Chintan Kapadia[3], Rahul Datta[4,*]

[1] Food Quality Testing Laboratory, N.M. College of Agriculture, Navsari Agricultural University, Navsari 396450, Gujarat, India

E-mail: susheelsingh@nau.in (S.S.); lokesh611991@gmail.com (L.K.S.); jyotinishad.nir@gmail.com (J.N.)

[2] Department of Natural Resource Management, College of Forestry, Navsari Agricultural University, Navsari 396450, Gujarat, India

E-mail: dileshwarnayak@jnu.ac.in (D.N.)

[3] Department of Plant Molecular Biology and Biotechnology, N.M. College of Agriculture, Navsari Agricultural University, Navsari 396450, Gujarat, India

E-mail: chintan_bt@nau.in (C.K.)

[4] Department of Geology and Pedology, Faculty of Forestry and Wood Technology, Mendel University in Brno, Zemedelska 1, 61300 Brno, Czech Republic

E-mail: rahulmedcure@gmail.com (R.D.)

***Corresponding Author:** Rahul Datta; E-mail: rahulmedcure@gmail.com;

SUPPORTING INFORMATION

The following supporting information is available free of charge at

Table S1.1. Weekly meteorological data for the 2022–23 growing season at the experimental site (Navsari, Gujarat, India).

Table S1.2. Weekly meteorological data for the 2023–24 growing season at the experimental site.

Table S2. Physicochemical properties of soil samples collected from the experimental site at Navsari Agricultural University.

Tables S3.1–S3.4. Year-wise residue data (mg kg^{-1}) for azoxystrobin and difenoconazole in turmeric leaves, soil, and rhizomes for 2022–23 and 2023–24 separately.

Table S4. Student's t-test results comparing DT_{50} values between year 1 (2022–23) and year 2 (2023–24) for all treatment combinations.

Figure S5. Representative LC-MS/MS chromatograms of azoxystrobin and difenoconazole in turmeric rhizomes at harvest, confirming residues below the LOQ.

Table S1.1 Weekly meteorological data for the 2022–23 growing season at the experimental site (Navsari, Gujarat, India).

Week	Temp Max (°C)	Temp Min (°C)	Mean Temp (°C)	RH Max (%)	RH Min (%)	Evap (mm/day)	Sunshine (hr/day)	Wind (km/hr)	Rainfall (mm/week)
27 May–2 June	33.90	27.60	30.75	83.3	61.2	5.40	6.50	9.20	0.0
3–9 Jun	34.60	27.10	30.85	84.6	61.0	4.30	8.50	7.00	0.0
10–16 Jun	33.10	25.50	29.30	93.0	67.6	3.10	7.00	4.90	68.0
17–23 Jun	31.80	24.00	27.90	95.5	78.9	2.30	4.40	6.10	78.0
24 Jun–30 Jun	32.80	24.50	28.65	93.7	71.2	2.40	5.00	6.10	64.0
1–7 Jul 2022	28.80	24.10	26.45	98.4	90.9	1.30	0.30	5.20	336.0
8–14 Jul	27.90	23.70	25.80	95.3	96.7	0.90	0.00	5.30	517.0
15–21 Jul	29.40	24.60	27.00	95.9	84.3	1.60	2.10	6.60	56.0
22–28 Jul	30.50	24.70	27.60	94.4	81.6	2.20	2.70	4.50	40.0
29 Jul–4 Aug	31.80	24.80	28.30	92.2	72.9	3.30	5.40	3.10	147.0
5–11 Aug	30.70	23.70	27.20	94.1	80.8	1.90	2.60	5.70	119.0
12–18 Aug	29.70	23.60	26.65	90.6	88.1	1.30	1.20	9.20	252.0
19–25 Aug	30.40	24.70	27.55	93.5	79.1	2.30	3.40	5.90	9.0
26 Aug–1 Sep	32.20	23.70	27.95	93.7	64.4	3.00	6.60	1.50	23.0
2–8 Sep	33.20	24.80	29.00	94.6	68.1	2.20	5.80	1.70	125.0
9–15 Sep	31.40	23.90	27.65	97.7	83.1	0.90	2.80	2.60	284.0
16–22 Sep	29.40	23.20	26.30	97.4	79.7	1.40	2.30	2.60	201.0
23–29 Sep	31.40	23.10	27.25	96.2	70.5	2.60	4.50	1.90	27.0
30 Sep–6 Oct	33.50	23.60	28.55	93.0	65.4	3.10	7.40	1.60	7.0
7–13 Oct	31.30	23.00	27.15	92.5	69.7	2.90	4.70	2.20	67.0
14–20 Oct	34.60	22.10	28.35	79.0	58.7	3.60	8.30	3.50	0.0
21–27 Oct	34.70	18.70	26.70	85.7	42.4	3.90	9.60	2.50	0.0
28 Oct–3 Nov	34.60	16.80	25.70	80.3	38.1	2.40	9.50	2.00	0.0
4–10 Nov	35.40	18.10	26.75	89.5	33.3	3.10	9.30	1.50	0.0
11–17 Nov	33.80	18.10	25.95	84.2	34.3	3.30	8.30	2.40	0.0
18–24 Nov	31.80	14.80	23.30	75.1	30.4	3.10	9.30	3.40	0.0
25 Nov–1 Dec	33.30	16.90	25.10	80.0	38.7	2.90	8.30	2.10	0.0
2–8 Dec	32.80	17.60	25.20	85.2	43.1	3.50	5.30	2.50	0.0
9–15 Dec	33.10	20.10	26.60	85.4	44.0	3.60	6.00	2.70	0.0
16–22 Dec	33.20	17.80	25.50	81.4	35.5	4.10	8.30	2.50	0.0
23–31 Dec	30.00	11.70	20.85	94.1	39.9	2.80	7.90	1.80	0.0

Week	Temp Max (°C)	Temp Min (°C)	Mean Temp (°C)	RH Max (%)	RH Min (%)	Evap (mm/day)	Sunshine (hr/day)	Wind (km/hr)	Rainfall (mm/week)
1–7 Jan 2023	29.10	15.20	22.15	80.1	40.7	2.60	5.90	5.31	0.0
8–14 Jan	29.80	12.80	21.30	82.9	34.9	3.00	7.40	2.78	0.0
15–21 Jan	29.30	10.90	20.10	88.1	38.4	3.30	8.60	2.12	0.0
22–28 Jan	27.50	12.70	20.10	82.9	35.9	3.00	5.30	3.78	0.0
29 Jan–4 Feb	30.80	16.70	23.75	85.7	43.0	3.50	4.20	4.56	0.0
5–11 Feb	33.60	13.60	23.60	86.5	29.5	3.20	9.40	2.43	0.0
12–18 Feb	35.10	13.50	24.30	75.0	19.9	3.10	10.00	3.08	0.0
19–25 Feb	36.10	13.70	24.90	87.4	24.5	4.40	9.40	2.16	0.0
26 Feb–4 Mar	35.90	15.40	25.65	87.8	26.8	3.90	9.10	2.01	0.0
5–11 Mar	36.30	18.60	27.45	66.1	31.5	3.70	5.90	3.59	0.0
12–18 Mar	36.10	20.00	28.05	71.5	39.3	4.10	4.20	3.75	0.3
19–25 Mar	30.90	19.00	24.95	94.1	56.0	3.10	7.30	3.67	0.0
26 Mar–1 Apr	32.30	19.80	26.05	94.9	44.2	3.00	8.20	3.66	0.0
2–8 Apr	34.30	22.90	28.60	89.1	41.0	5.10	8.50	3.46	0.0
**Number of weeks (N)**	**45**								
**Seasonal mean**	**32.27**	**20.12**	**26.20**	**88.04**	**54.67**	**2.97**	**6.15**	**3.69**	**53.78 (mean/week)**
								**Total rainfall (season)**	**2420.30 mm**

Table S1.2 Weekly meteorological data for the 2023–24 growing season at the experimental site.

Week	Temp Max (°C)	Temp Min (°C)	Mean Temp (°C)	RH Max (%)	RH Min (%)	Evap (mm/day)	Sunshine (hr/day)	Wind (km/hr)	Rainfall (mm/week)
28 May–3 June	36.40	26.70	31.55	79.8	54.2	5.50	10.00	5.75	0.0
4–10 Jun	34.60	28.10	31.35	78.0	59.3	5.60	8.00	6.98	0.0
11–17 Jun	34.80	27.20	31.00	80.3	65.1	3.90	7.50	13.45	13.0
18–24 Jun	33.90	27.90	30.90	82.2	65.6	5.50	6.10	8.03	0.0
25 Jun–1 Jul	29.30	23.90	26.60	98.1	93.2	1.00	0.30	3.96	384.0
2–8 Jul 2023	30.80	24.90	27.85	93.7	83.8	1.20	2.30	3.49	254.2
9–15 Jul	28.80	24.50	26.65	98.0	94.4	1.60	0.40	4.78	167.5
16–22 Jul	29.80	25.30	27.05	97.0	91.2	1.30	0.80	3.46	72.0
23–29 Jul	29.20	24.70	26.95	98.4	87.0	0.90	0.30	5.15	517.0
30 Jul–5 Aug	29.70	25.20	27.45	90.6	87.5	1.10	0.80	8.09	41.0
6–12 Aug	30.50	25.70	28.10	89.0	77.1	1.20	1.20	7.68	11.0
13–19 Aug	30.10	25.40	27.75	90.9	77.1	1.60	3.80	6.15	4.0
20–26 Aug	30.90	24.90	27.90	94.2	78.3	1.50	2.70	4.32	12.0
27 Aug–2 Sep	32.30	24.40	28.35	93.2	58.4	2.80	8.30	2.69	1.0
3–9 Sep	32.00	24.20	28.10	92.1	70.0	2.50	3.70	1.63	57.0
10–16 Sep	31.50	25.00	28.25	94.4	74.4	2.30	6.80	3.06	16.0
17–23 Sep	30.40	24.50	27.45	96.2	83.3	2.10	2.30	3.07	93.0
24–30 Sep	32.70	23.40	28.05	95.9	71.8	1.50	4.30	1.04	123.0
1–7 Oct	33.80	23.90	28.85	94.2	65.6	3.40	7.80	1.00	0.0
8–14 Oct	34.90	23.20	29.05	97.0	57.7	3.60	8.10	0.43	0.0
15–21 Oct	35.60	22.30	28.95	92.4	43.1	3.40	8.00	0.79	0.0
22–28 Oct	36.10	19.60	27.85	87.5	34.6	3.50	8.50	0.49	0.0
29 Oct–4 Nov	35.30	18.10	26.70	81.9	30.8	4.40	9.30	1.02	0.0
5–11 Nov	35.50	20.30	27.90	76.4	36.6	4.00	8.10	0.98	0.0
12–18 Nov	34.50	18.90	26.70	75.1	37.6	4.00	8.10	1.71	0.0
19–25 Nov	34.10	18.70	26.40	82.3	39.0	3.60	6.70	1.56	0.0
26 Nov–2 Dec	28.30	19.20	23.75	95.2	72.8	1.90	3.90	1.91	42.0
3–9 Dec	31.10	19.90	25.50	96.0	61.0	2.70	5.70	2.59	0.0
10–16 Dec	31.70	16.70	24.20	85.7	40.2	4.30	7.70	1.26	0.0
17–23 Dec	29.40	17.80	23.60	66.8	36.9	4.20	4.10	3.90	0.0
24–31 Dec 23	32.20	13.60	22.90	94.6	39.2	3.60	7.70	1.02	0.0
Jan 2024 (1–7)	29.20	15.50	22.35	94.0	57.0	0.00	0.00	4.80	0.0
8–14 Jan	31.30	17.70	24.50	95.0	48.0	0.00	0.00	4.60	0.0
15–21 Jan	29.40	11.80	20.60	94.0	42.0	0.00	0.00	7.90	0.0
22–28 Jan	30.70	11.60	21.15	82.0	25.0	0.00	0.00	9.10	0.0
29 Jan–4 Feb	31.50	14.10	22.80	95.0	39.0	0.00	0.00	8.50	0.0
5–11 Feb	33.20	14.80	24.00	88.0	35.0	0.00	0.00	8.90	0.0

Week	Temp Max (°C)	Temp Min (°C)	Mean Temp (°C)	RH Max (%)	RH Min (%)	Evap (mm/day)	Sunshine (hr/day)	Wind (km/hr)	Rainfall (mm/week)
12–18 Feb	32.70	15.50	24.10	59.0	60.0	0.00	0.00	7.90	0.0
19–25 Feb	31.60	13.90	22.75	92.0	32.0	0.00	0.00	9.80	0.0
26 Feb–4 Mar	34.00	17.10	25.55	86.0	39.0	0.00	0.00	8.80	0.0
5–11 Mar	32.00	12.20	22.10	85.0	25.0	0.00	0.00	10.00	0.0
12–18 Mar	34.30	16.40	25.35	90.0	36.0	0.30	0.00	9.20	0.0
19–25 Mar	34.70	17.10	25.90	92.0	37.0	0.00	0.00	8.50	0.0
**Number of weeks (N)**	**43**								
**Seasonal mean**	**32.20**	**20.60**	**26.40**	**88.80**	**56.78**	**2.09**	**3.80**	**4.87**	**42.02 (mean/week)**
**Total rainfall (season)**									**1807.70 mm**

Table S2. Physicochemical properties of soil samples collected from the experimental site at Navsari Agricultural University

Sr. No.	Soil Property	Unit	2022-23	2023-24	Analytical Method	Reference
**A**	**Physical Property**					
1	Soil Texture	-	Clayey	Clayey	International Pipette Method	Piper (1966)
**B**	**Chemical Properties**					
2	Soil pH (1:2.5)	-	8.01	7.98	Potentiometric method	Jackson (1973)
3	EC (1:2.5)	dS m^{-1}	0.32	0.35	Conductometry	Jackson (1973)
4	Organic Carbon	%	0.36	0.42	Walkley and Black's wet oxidation	Walkley and Black (1934)
**C**	**Available Nutrients**					
5	Available Nitrogen	kgha^{-1}	204.4	214.1	Alkaline permanganate method	Subbiah and Asija (1956)
6	Available Phosphorus	kg ha^{-1}	36.2	34.7	Olsen's method (0.5 M $NaHCO_3$)	Olsen *et al.* (1954)
7	Available Potassium	kg ha^{-1}	300.3	312.4	Flame photometry (Neutral N NH_4OAc)	Jackson (1973)

References :
Jackson, M. L. (1973). Soil Chemical Analysis. Prentice Hall of India Pvt. Ltd., New Delhi.

Olsen, S. R., Cole, C. V., Watanabe, F. S., & Dean, L. A. (1954). Estimation of available phosphorus in soils by extraction with sodium bicarbonate. USDA Circular No. 939.

Piper, C. S. (1966). Soil and Plant Analysis. Hans Publishers, Bombay.

Subbiah, B. V., & Asija, G. L. (1956). A rapid procedure for the determination of available nitrogen in soils. Current Science, 25(8), 259-260.

Walkley, A., & Black, I. A. (1934). An examination of the Degtjareff method for determining soil organic matter, and a proposed modification of the chromic acid titration method. Soil Science, 37(1), 29-38.

Table S3.1 :- Residues and dissipation pattern of azoxystrobin in/on Turmeric leaves

DAA	Residue (mg/kg)											
	2022-23						2023-24					
	RD (150 g a.i/ha)			2RD (300 g a.i/ha)			RD (150 g a.i/ha)			2RD (300 g a.i/ha)		
	R1	R2	R3	R1	R2	R3	R1	R2	R3	R1	R2	R3
0 (hrs)	0.164	0.159	0.163	0.279	0.291	0.298	0.198	0.194	0.199	0.328	0.333	0.334
1	0.133	0.139	0.137	0.263	0.278	0.271	0.192	0.191	0.187	0.311	0.314	0.301
3	0.130	0.125	0.134	0.240	0.249	0.255	0.178	0.183	0.171	0.293	0.299	0.287
5	0.116	0.109	0.114	0.202	0.199	0.200	0.142	0.133	0.139	0.265	0.254	0.240
7	0.096	0.099	0.097	0.176	0.181	0.178	0.117	0.121	0.118	0.185	0.191	0.187
10	0.055	0.064	0.056	0.161	0.157	0.152	0.076	0.078	0.080	0.156	0..163	0.150
15	0.022	0.038	0.044	0.090	0.088	0.079	0.051	0.046	0.054	0.087	0.089	0.085
30	0.011	0.019	0.020	0.041	0.040	0.051	0.022	0.026	0.021	0.030	0.034	0.033
60	0.010	0.012	0.015	0.028	0.031	0.029	0.010	0.006	0.007	0.009	0.009	0.011
90	BQL	BQL	BQL	BQL	BQL	BQL	BQL	BQL	BQL	BQL	BQL	BQL

LOQ :0.010 mg kg^{-1} for azoxystrobin

Table S3.2 :- Residues and dissipation pattern of azoxystrobin in soil

DAA	Residue (mg/kg)											
	2022-23						2023-24					
	RD (150 g a.i/ha)			2RD (300 g a.i/ha)			RD (150 g a.i/ha)			2RD (300 g a.i/ha)		
	R1	R2	R3	R1	R2	R3	R1	R2	R3	R1	R2	R3
0 (hrs)	0.249	0.237	0.226	0.387	0.398	0.380	0.234	0.233	0.248	0.382	0.380	0.389
1	0.239	0.228	0.221	0.375	0.381	0.371	0.220	0.211	0.210	0.372	0.357	0.355
3	0.201	0.187	0.189	0.338	0.314	0.318	0.189	0.176	0.178	0.349	0.351	0.350
5	0.101	0.089	0.091	0.241	0.250	0.253	0.125	0.131	0.124	0.235	0.248	0.241
7	0.097	0.078	0.088	0.202	0.211	0.204	0.091	0.073	0.083	0.211	0.224	0.229
10	0.091	0.074	0.081	0.153	0.124	0.136	0.086	0.070	0.076	0.145	0.158	0.168
15	0.089	0.071	0.079	0.150	0.119	0.133	0.074	0.078	0.068	0.125	0.132	0.115
30	0.018	0.029	0.016	0.068	0.061	0.071	0.039	0.041	0.045	0.084	0.079	0.080
60	0.015	0.019	0.021	0.035	0.039	0.040	0.029	0.024	0.020	0.049	0.041	0.033
90	0.008	0.007	0.010	0.020	0.022	0.027	0.011	0.017	0.012	0.013	0.016	0.019

LOQ :0.010 mg kg^{-1} for azoxystrobin

Table S3.3 :- Residues and dissipation pattern of difenoconazole in/on turmeric leaves

DAA	Residue (mg/kg)											
	2022-23						2023-24					
	RD (150 g a.i/ha)			2RD (300 g a.i/ha)			RD (150 g a.i/ha)			2RD (300 g a.i/ha)		
	R1	R2	R3	R1	R2	R3	R1	R2	R3	R1	R2	R3
0 (hrs)	0.241	0.250	0.239	0.397	0.350	0.385	0.278	0.275	0.283	0.390	0.333	0.325
1	0.170	0.169	0.158	0.248	0.257	0.251	0.196	0.189	0.199	0.301	0.300	0.321
3	0.168	0.157	0.160	0.235	0.220	0.224	0.185	0.173	0.176	0.278	0.288	0.271
5	0.163	0.149	0.151	0.228	0.209	0.211	0.179	0.164	0.166	0.269	0.246	0.249
7	0.160	0.147	0.150	0.124	0.118	0.111	0.146	0.132	0.145	0.219	0.198	0.218
10	0.034	0.038	0.053	0.048	0.053	0.074	0.042	0.058	0.069	0.123	0.117	0.121
15	0.024	0.022	0.031	0.034	0.031	0.043	0.034	0.032	0.029	0.089	0.092	0.094
30	0.011	0.008	0.012	0.015	0.011	0.017	0.029	0.021	0.031	0.030	0.031	0.047
60	0.006	0.005	0.007	0.008	0.009	0.010	0.011	0.009	0.013	0.010	0.014	0.018
90	BQL	BQL	BQL	BQL	BQL	BQL	BQL	BQL	BQL	BQL	BQL	BQL

LOQ : 0.005 mg kg^{-1} for difenoconazole

Table S3.4 :- Residues and dissipation pattern of difenoconazole in soil

DAA	Residue (mg/kg)											
	2022-23						2023-24					
	RD (150 g a.i/ha)			2RD (300 g a.i/ha)			RD (150 g a.i/ha)			2RD (300 g a.i/ha)		
	R1	R2	R3	R1	R2	R3	R1	R2	R3	R1	R2	R3
0 (hrs)	0.041	0.037	0.035	0.071	0.073	0.068	0.062	0.068	0.059	0.098	0.096	0.101
1	0.039	0.037	0.034	0.069	0.065	0.067	0.051	0.048	0.044	0.087	0.082	0.079
3	0.038	0.036	0.033	0.063	0.060	0.055	0.049	0.047	0.043	0.084	0.080	0.073
5	0.037	0.033	0.031	0.062	0.055	0.052	0.048	0.043	0.040	0.082	0.073	0.069
7	0.030	0.026	0.025	0.050	0.043	0.042	0.039	0.034	0.033	0.067	0.068	0.066
10	0.027	0.022	0.023	0.045	0.037	0.038	0.035	0.029	0.030	0.059	0.069	0.061
15	0.020	0.021	0.014	0.040	0.034	0.032	0.026	0.027	0.028	0.034	0.042	0.039
30	0.017	0.018	0.010	0.028	0.030	0.026	0.022	0.023	0.021	0.021	0.028	0.026
60	0.008	0.010	0.007	0.010	0.018	0.012	0.009	0.008	0.012	0.016	0.014	0.020
90	BQL	BQL	BQL	BQL	BQL	BQL	BQL	BQL	BQL	BQL	BQL	BQL

LOQ : 0.005 mg kg^{-1} for difenoconazole

Table S4. **Student's t-test results comparing DT_{50} values between year 1 (2022–23) and year 2 (2023–24) for all treatment combinations.**

Compound	Matrix	Dose	DT_{50} 2022–23 (days)	DT_{50} 2023–24 (days)	Difference (days)	SE_pooled	t-statistic	df	p-value	Significance	Pooling
**Azoxystrobin**	Leaves	RD	18.31	16.07	+2.24	0.3785	0.8129	16	**0.428**	**ns (p > 0.05)**	Justified
**Azoxystrobin**	Leaves	2×RD	16.39	14.08	+2.31	0.2285	1.3992	16	**0.181**	**ns (p > 0.05)**	Justified
**Azoxystrobin**	Soil	RD	19.36	23.30	−3.94	0.6505	−0.9566	16	**0.353**	**ns (p > 0.05)**	Justified
**Azoxystrobin**	Soil	2×RD	22.32	20.51	+1.81	0.4660	0.5804	16	**0.570**	**ns (p > 0.05)**	Justified
**Difenoconazole**	Leaves	RD	13.79	14.39	−0.60	0.2592	−0.2313	16	**0.820**	**ns (p > 0.05)**	Justified
**Difenoconazole**	Leaves	2×RD	13.30	13.01	+0.29	0.1954	0.1483	16	**0.884**	**ns (p > 0.05)**	Justified
**Difenoconazole**	Soil	RD	24.31	22.01	+2.30	0.2833	1.2012	16	**0.247**	**ns (p > 0.05)**	Justified
**Difenoconazole**	Soil	2×RD	20.74	19.01	+1.73	0.2982	0.8721	16	**0.396**	**ns (p > 0.05)**	Justified

Two-tailed t-test, $\alpha = 0.05$. SE_pooled = $\sqrt{(SE^2\_DT_{50}\_Y1 + SE^2\_DT_{50}\_Y2)}$. df = 16 ($n_1+n_2-4$). ns = not significant. Green = $p > 0.05$ (pooling justified); Red = $p < 0.05$.

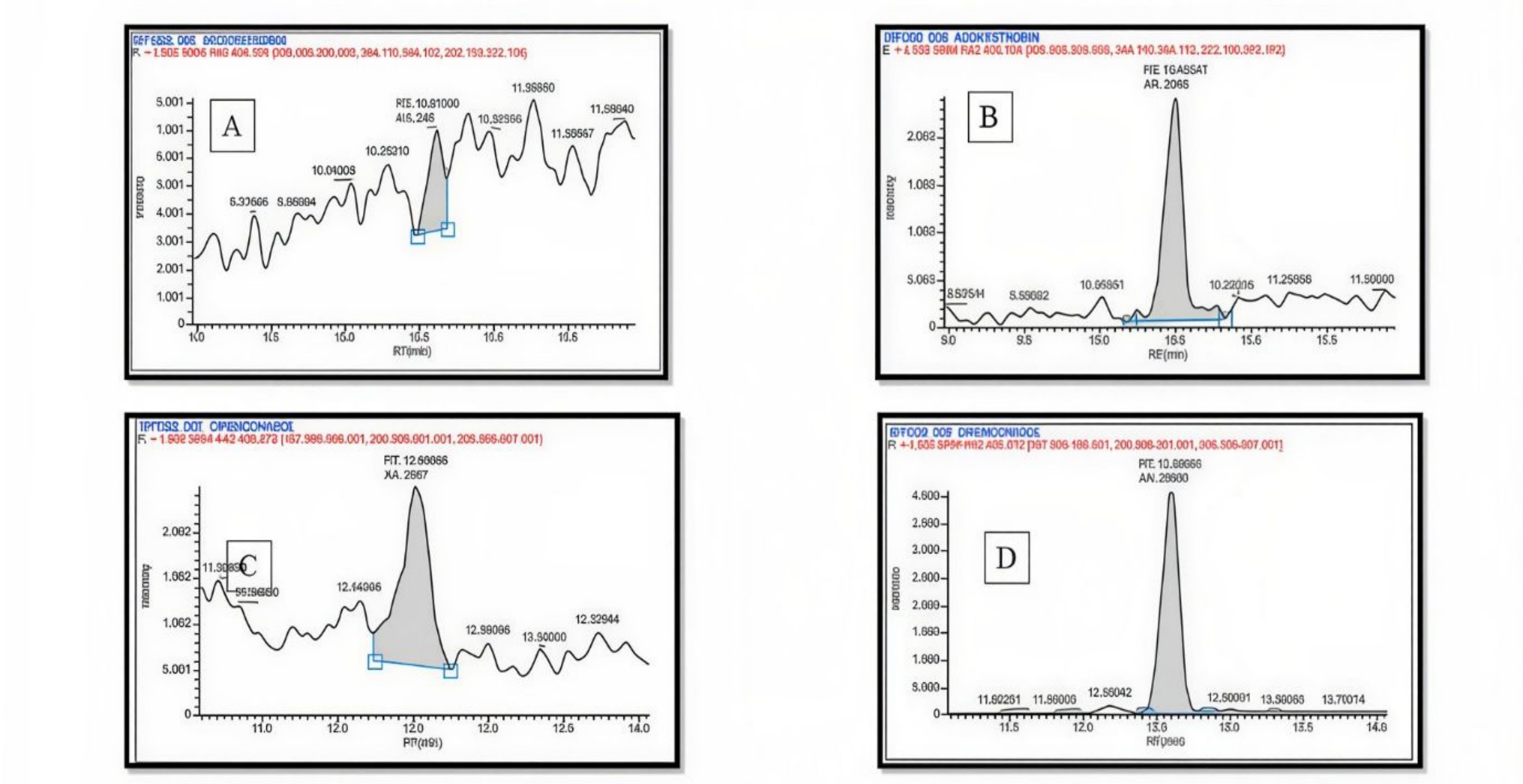

Figure S5. Representative LC-MS/MS chromatograms of azoxystrobin and difenoconazole in turmeric rhizomes at harvest, confirming residues below the LOQ. A: Turmeric rhizome (2XRD), B: Azoxystrobin (2.5µg/L), C: Turmeric rhizome (2XRD), D: Difenoconazole (2.5µg/L)
